# Significant correlation between plasma proteome profile and pain intensity, sensitivity, and psychological distress in women with fibromyalgia

**DOI:** 10.1038/s41598-020-69422-z

**Published:** 2020-07-27

**Authors:** Karin Wåhlén, Malin Ernberg, Eva Kosek, Kaisa Mannerkorpi, Björn Gerdle, Bijar Ghafouri

**Affiliations:** 1 Pain and Rehabilitation Center, and Department of Health, Medicine and Caring Sciences, Linköping University, Linköping, Sweden; 2 Department of Dental Medicine, Karolinska Institutet and Scandinavian Center for Orofacial Neurosciences (SCON), 141 04 Huddinge, Sweden; 3 Department of Clinical Neuroscience, Karolinska Institutet, 171 77 Stockholm, Sweden; 4 Department of Health and Rehabilitation/Physiotherapy, Institute of Neuroscience and Physiology, Sahlgrenska Academy, Gothenburg University, Gothenburg, Sweden

**Keywords:** Proteomics, Pain, Musculoskeletal system, Inflammation, Biomarkers, Anxiety, Depression, Fibromyalgia

## Abstract

Fibromyalgia (FM) is a complex pain condition where the pathophysiological and molecular mechanisms are not fully elucidated. The primary aim of this study was to investigate the plasma proteome profile in women with FM compared to controls. The secondary aim was to investigate if plasma protein patterns correlate with the clinical variables pain intensity, sensitivity, and psychological distress. Clinical variables/background data were retrieved through questionnaires. Pressure pain thresholds (PPT) were assessed using an algometer. The plasma proteome profile of FM (n = 30) and controls (n = 32) was analyzed using two-dimensional gel electrophoresis and mass spectrometry. Quantified proteins were analyzed regarding group differences, and correlations to clinical parameters in FM, using multivariate statistics. Clear significant differences between FM and controls were found in proteins involved in inflammatory, metabolic, and immunity processes. Pain intensity, PPT, and psychological distress in FM had associations with specific plasma proteins involved in blood coagulation, metabolic, inflammation and immunity processes. This study further confirms that systemic differences in protein expression exist in women with FM compared to controls and that altered levels of specific plasma proteins are associated with different clinical parameters.

## Introduction

Fibromyalgia (FM) is a chronic complex pain condition with an estimated prevalence of about 1–4% in the general population^1,2^. Key cardinal characteristics in FM are generalized widespread pain and hyperalgesia/allodynia^3^, and symptoms as fatigue, depression, anxiety, sleep problems, cognitive impairments, headache, and muscle tenderness are often present. FM is more common in women (approximately six out of ten patients in unbiased studies^4^), where factors such as increasing age and obesity could play a role^5–7^. Over the past three decades the American College of Rheumatology (ACR) have released several criteria to assess a clinical diagnosis of FM. In the 1990 version^3^, diagnosis is based on semi-objective examination of hyperalgesia (tender points), presence of generalized pain, in combination with evaluation of anamnestic reports. In the criteria from 2010/2011 and 2016^8–10^, diagnosis is based upon physician-based questionnaires defining widespread pain and symptom severity; a medical examination of hyperalgesia is no longer needed. Generally, there is a lack of mechanism-based diagnoses within FM and other chronic pain conditions since the pathophysiological and molecular mechanisms behind it are insufficiently elucidated. Hence, there is a need for valid objective biological markers (biomarkers) not only for diagnosis, but for evaluation of treatments and other interventions.

There is increasing evidence that not only central mechanisms are essential for the presentation and the maintenance of the clinical picture in FM patients. Hence, in addition to the studies reporting central alterations (e.g., anatomical and functional changes in the brain, activation of glia cells, opioidergic dysregulation, impaired top-down modulation and nociception-driven amplification of neural signalling), several studies have shown systemic and peripheral changes in FM, e.g., regarding cytokine profile^11–13^, inflammatory lipids^14^, alteration of different metabolites and changes of the gut microbiota^15–17^, and altered protein changes in muscles^18–20^. So far, no clinically validated biomarkers for FM have been established. Body fluids such as plasma, saliva, urine, and cerebrospinal fluid (CSF) have been investigated for the study of protein biomarkers in different chronic pain conditions e.g., neuropathic pain^21–23^, chronic widespread pain (CWP)^24–28^, and FM^11,15,23,29–34^. Metabolic and proteomic analysis of these biofluids, especially the metabolomics of blood, urine, and gut microbiome composition^15,17^, as well as the protein profile of plasma and CSF^11,27^, have been reported to be able to discriminate FM/CWP patients from healthy controls. Further, most reported biomarker studies in FM/CWP point towards an inflammatory state/response in these chronic pain patients. However, the results are not unequivocally consistent; in one of the largest studies of FM (n = 39), only moderate differences were found in the CSF proteome between patients and controls^34^.

The plasma proteome has been of large interest when investigating biomarkers in different diseases. The advantages of studying plasma for biomarker discovery are that it is easy to access, low-invasive to the subject, and rich in proteins. Furthermore, the blood is in direct contact with all surrounding tissue and therefore has the potential to reflect both peripheral tissue and systemic changes. In a preliminary study of the serum proteome in FM, Ruggiero et al*.* found upregulated levels of the proteins alpha-1-antitrypsin, transthyretin, and retinol-binding protein 4 compared to healthy controls^33^. More recently, Ramíres-Tejero et al*.* explored the plasma proteome in FM compared to controls and reported significant changes in several proteins involved in inflammation, suggesting haptoglobin and fibrinogen as potential biomarkers for FM^31^. Both these studies are in line with our previous proteomic study of plasma in CWP patients (mainly FM) compared to healthy controls and its correlation to pain intensity and psychological distress^27,28^. However, these studies were performed on small cohorts of patients, and the validity of the identified protein changes needs to be investigated in a larger cohort.

Due to the limited numbers of studies exploring the plasma proteome profile in FM, including their low sample sizes, more studies are warranted to cover the existing knowledge gap concerning the activated molecular mechanisms in FM. This also includes efforts to identify potential biomarkers for FM, including aspects of the clinical presentation. Therefore, the primary aim of this study was to investigate the plasma proteome profile in FM patients compared to healthy subjects (CON). The secondary aim was to investigate if plasma protein patterns correlate with the clinical variables pain intensity, pain sensitivity, and psychological distress.

## Results

### Background data

No differences in age were found between the groups. However, body mass index (BMI) was significantly higher in FM compared to CON; overweight (i.e., BMI ≥ 25 kg/m^2^) was present in 18 patients and eight controls. Pressure pain thresholds (PPT) were significantly lower, while the pain intensity (visual analogue scale, VAS), the scores from the fibromyalgia impact questionnaire (FIQ), and the hospital anxiety and depression scale (HADS) were significantly higher in FM compared to CON (Table 1).

**Table 1:** Background data.

Variables	CON (n = 31)	FM (n = 30)	*p* value*
	Median (interquartile range)	Median (interquartile range)	
Age (years)	57 (9)	54 (13)	0.366
BMI (kg/m^2^)	24 (3)	26 (6)	< 0.05
FM duration (years)	–	12 (14)	–
Tender points (number)	–	16 (2)	–
Pain intensity (VAS)	0 (2)	50 (36)	< 0.001
PPT all sites (kPa)	340 (146)	175 (88)	< 0.001
FIQ total	2 (8)	58 (24)	< 0.001
HADS total (psychological distress)	3 (5)	13 (14)	< 0.001

*FM* Fibromyalgia, *CON* controls, *BMI* body mass index, *VAS* visual analogue scale, *PPT* pressure pain thresholds, *FIQ* fibromyalgia impact questionnaire, *HADS* Hospital Anxiety and Depression Scale.

*Mann Whitney-*U* statistical test was used for all variables.

### Proteomics

A total of 381 proteins including different proteoforms were detected and matched in the two-dimensional gel electrophoresis (2-DE) analysis and used for multivariate modeling. A representative 2-DE gel of the identified plasma proteins, including mass spectrometry (MS) data from each multivariate data analysis (MVDA) model is provided in Supplementary Figure S1 and Supplementary Table S1.

## MVDA models

### Group difference in plasma proteome (FM vs. CON)

**Figure 1:**
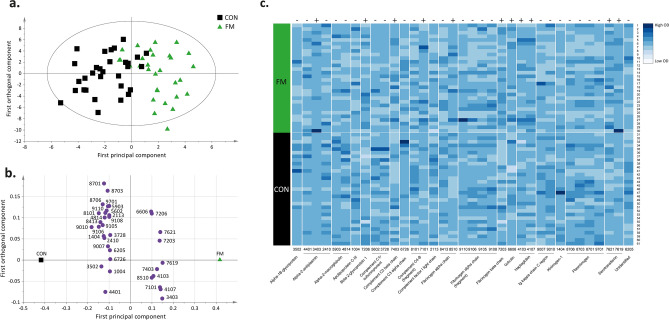
Differences in plasma proteins between FM and CON using OPLS-DA modeling. (**a**) Score plot showing the separation between the FM and CON group. (**b**) Loading plot displaying proteoforms with VIP > 1. Numbers in the loading plot refers to equal spot numbers in (**c**), Table 2 and in supplementary Figure S1 and Table S1. (**c**) Heatmap showing individual (from each participant) optical density (OD) of quantified proteins for each proteoform with a VIP > 1 between FM and CON. The darker the color intensity, the higher OD of the proteoform (see color key). The loading and score plots were created in SIMCA P + (version 15), and the heatmap was created in R using the gplots package^74^. +, upregulated in FM compared to CON; —, downregulated in FM compared to CON.

The plasma proteome from FM and CON was compared in an orthogonal partial least square—discriminant analysis (OPLS-DA) model (Fig. 1a, b). The significant OPLS-DA model had one predictive component and one orthogonal component, which showed a high explained variation, predictivity, and a significant CV-ANOVA (R^2^ = 0.61, Q^2^ = 0.36, CV-ANOVA: *p* < 0.001). The score plot showed a clear separation between FM and CON (Fig. 1a), and the loading plot showed 18 unique proteins, expressed as 35 proteoforms, that were significantly altered (variable influence on projection (VIP) > 1) and were able to discriminate between FM and CON (Fig. 1b). Differences in the optical density of quantified proteins between the two groups are shown in Fig. 1c. The proteoforms with highest VIP values (VIP > 1.5), which contributed most to the separation between the two groups were upregulated proteoforms of serotransferrin (spot number 7619), alpha-2-antiplasmin (spot number 3403), haptoglobin (spot number 4107), and downregulated proteoforms of Ig kappa chain C region (spot number 9010), complement C4-B (fragment) (spot number 8101), and fibrinogen alpha chain (fragment) (spot number 9106) (Table 2). Out of the 35 significant proteoforms, 11 were positively associated with the FM group, all upregulated proteoforms of serotransferrin (spot number 7619 and 7621), alpha-2-antiplasmin (spot number 3403), haptoglobin (spot number 4107 and 4103), fibrinogen alpha chain (8510), fibrinogen beta chain (spot number 7203), complement C4-B (fragment) (spot number 7101), complement C3 beta chain (spot number 7403), beta-2-glycoprotein 1 (spot number 7206), and gelsolin (spot number 6606) (Fig. 1b, c, Table 2).

**Table 2:** Significant plasma proteins in the OPLS-DA model of FM and CON.

Spot number	Protein name	Accession number	VIPpred	p(corr)	Fold change FM versus CON
9010	Ig kappa chain C region	P01834	1.92	− 0.56	0.40
7619	Serotransferrin	P02787	1.61	+ 0.47	1.50
3403	Alpha-2-antiplasmin	P08697	1.58	+ 0.46	2.04
8101	Complement C4-B (fragment)	P0C0L5	1.57	− 0.45	0.71
9106	Fibrinogen alpha chain (fragment)	P02671	1.57	− 0.45	0.27
4107	Haptoglobin	P00738	1.51	+ 0.44	1.26
8413	Fibrinogen alpha chain	P02671	1.49	− 0.43	0.34
7621	Serotransferrin	P02787	1.48	+ 0.43	1.17
7101	Complement C4-B (fragment)	P0C0L5	1.48	+ 0.43	1.27
7203*	Fibrinogen beta chain	P02675	1.45	+ 0.42	1.59
3502	Alpha-1B-glycoprotein	P04217	1.43	− 0.41	0.87
9105	Fibrinogen alpha chain (fragment)	P02671	1.37	− 0.40	0.55
8706	Plasminogen	P00747	1.37	− 0.40	0.84
7403	Complement C3 beta chain	P01024	1.33	+ 0.39	1.19
1404*	Kininogen-1	P01042	1.33	− 0.38	0.72
8701	Plasminogen	P00747	1.30	− 0.38	0.94
2410*	Alpha-2-antiplasmin	P08697	1.28	− 0.37	0.66
4814	Alpha-2-macroglobulin	P01023	1.28	− 0.37	0.41
4401	Alpha-2-antiplasmin	P08697	1.24	− 0.36	0.84
9110	Fibrinogen alpha chain (fragment)	P02671	1.24	− 0.36	0.44
5602	Complement C1r subcomponent	P00736	1.17	− 0.34	0.85
4103	Haptoglobin	P00738	1.14	+ 0.33	1.24
8703	Plasminogen	P00747	1.12	− 0.33	0.91
9701	Plasminogen	P00747	1.11	− 0.32	0.74
9007**	Ig kappa chain C region	P01834	1.11	− 0.32	0.62
7206	Beta-2-glycoprotein 1	P02749	1.06	+ 0.31	1.10
8510	Fibrinogen alpha chain	P02671	1.06	+ 0.31	1.19
9108	Fibrinogen alpha chain (fragment)	P02671	1.05	− 0.30	0.26
5903	Alpha-2-macroglobulin	P01023	1.05	− 0.30	0.65
2113	Complement factor I light chain	P05156	1.05	− 0.30	0.88
6606	Gelsolin	P06396	1.03	+ 0.30	1.01
1004	Apolipoprotein C-III	P02656	1.01	− 0.29	0.33
6205	Unidentified	Unknown	1.01	− 0.29	0.91
6726*	Complement C3b alpha chain	P01024	1.00	− 0.29	0.73
3728	Complement C1r subcomponent	P00736	1.00	− 0.29	0.74

Spot numbers refers to equal numbers in Fig. 1b, c and marked proteins spots in supplementary Figure S1 and Table S1. Accession numbers are according to the protein data base UniProt (www.uniprot.org). Variable influence on projections (VIP) indicates the importance of the regressor, the higher value, the more important for the model. A VIP > 1 is considered as a significant protein, and here, only the significant proteins are shown. p(corr) is shown and is the multivariate correlation coefficient of each variable (protein) for the model. FM is coded 1, and CON coded 0. Hence, a positive p(corr) for a variable indicates a positive multivariate correlation versus FM belonging, and a negative p(corr), indicates a negative multivariate correlation with FM. For CON, the relationship is reversed. Please note that fold change is a univariate measure and do not necessarily correspond to p(corr); i.e., a positive fold change (FM > CON), is not automatically equal to a positive p(corr).

*Indicates shared proteoform in the model of PPT in FM, **indicated shared proteoform in the model of HADS in FM.

#### Pathway analysis of proteins associated with group differences FM and CON

**Figure 2:**
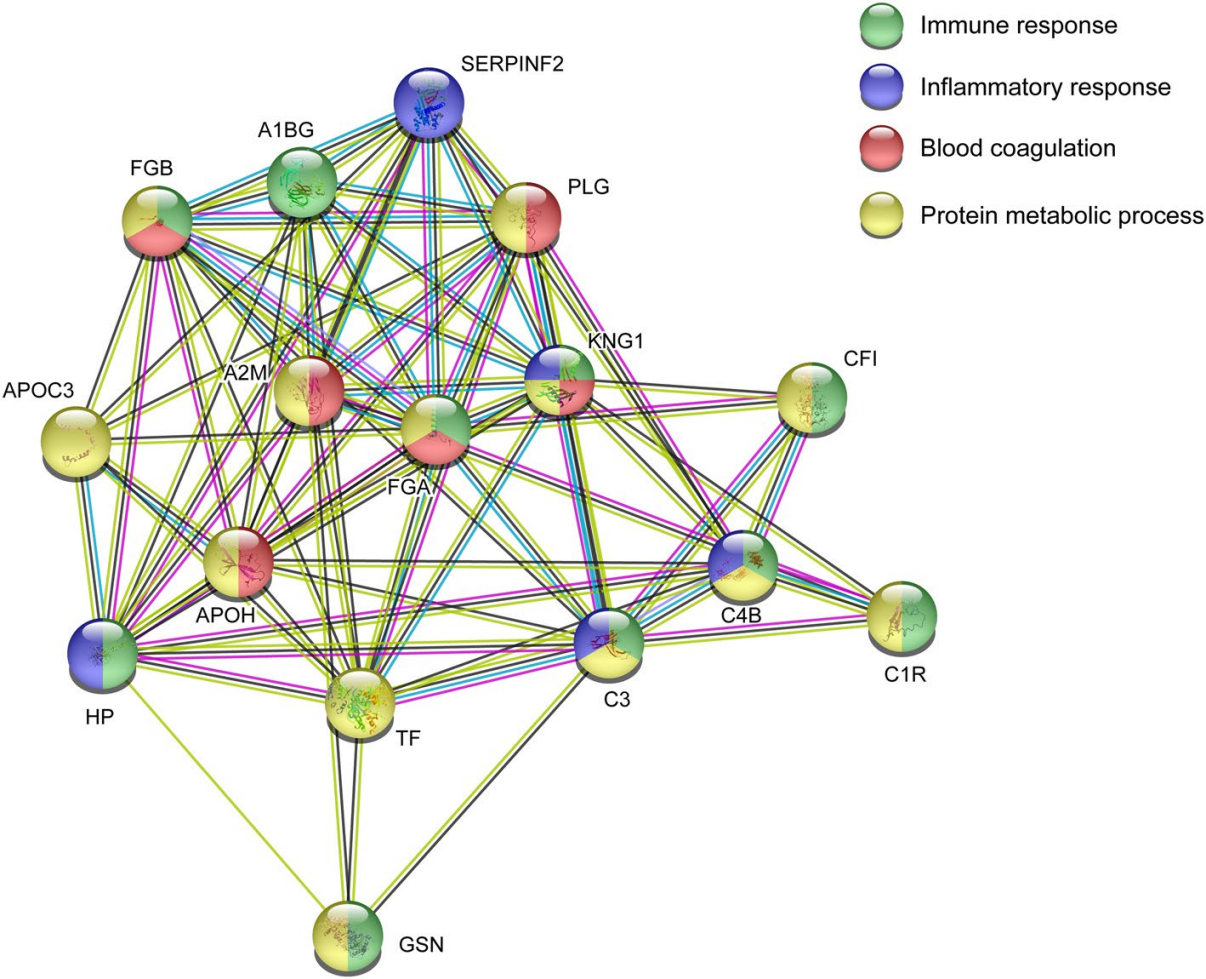
Pathways analysis group differences FM and CON. Investigation of functional protein network from significant plasma proteins that could discriminate between FM and CON. The STRING version 11 was used to create the network analysis (https://string-db.org/). *A2M* Alpha-2-macroglobulin, *GSN* Gelsolin, *HP* Haptoglobin, *FGA* Fibrinogen alpha chain, *FGB* Fibrinogen beta chain, *APOH* Beta-2-glycoprotein 1, *PLG* Plasminogen, *SERPINF2* Alpha-2-antiplasmin, *KNG1* Kininogen-1, *CFI* Complement factor I, *A1BG* Alpha-1B-glycoprotein, *C3* Complement C3, *TF* Serotransferrin, *APOC3* Apolipoprotein C-III, *C1R* Complement C1r subcomponent, *C4B* Complement C4-B.

To investigate functional protein interactions, the discriminant proteins (Fig. 1, Table 2) were analyzed using Search Tool for Retrieval of Interacting Genes/Proteins (STRING) pathway analysis (Fig. 2). Sixteen of 18 proteins were included in the identified network. Immunoglobulin kappa chain C region and the unidentified protein were not identified by the search engine. The colored lines represent different types of evidence of the associated interaction between the proteins in the network, e.g., complement C3 is closely interacted with the other complement proteins, but also with fibrinogen alpha chain, serotransferrin, kininogen-1, and haptoglobin. Gelsolin is located further away in the network and has indirect interactions with the other complement proteins through complement C3, haptoglobin, serotransferrin, and alpha-2-maroglobulin. In this enriched network, several biological processes were identified, i.e., immune response (FDR = 1.23E−06), inflammatory response (FDR = 0.00026), blood coagulation (FDR = 1.58E−06), and protein metabolic processes (FDR = 8.39E−06). The proteins positively associated with FM were proteins belonging to immune and inflammatory processes.

### Pain intensity in FM

**Figure 3:**
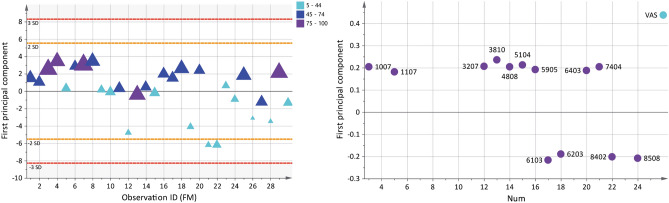
Pain intensity and associated plasma proteins in FM. The Score plot (left) shows a within-group separation in the FM group based on pain intensity (visual analogue scale, VAS). The plot shows that FM patients are grouped as having mild (5–44), moderate (45–74), and severe (75–100) pain intensity. The larger the triangle, the higher the pain intensity. The loading plot (right) shows 13 significant proteoforms (VIP > 1) associated with pain intensity. Nine proteoforms were associated with higher pain intensity in FM (VAS > 45). Numbers in the loading plot equals the spot numbers in Table 3 and supplementary Figure S1 and Table S1. The loading and score plots were created in SIMCA P + (version 15).

In the OPLS model of pain intensity (VAS), 11 unique proteins, expressed as 13 proteoforms were multivariate correlated with pain intensity in FM (Fig. 3). The significant model had one principal component and showed a good explained variation, predictivity, and a significant CV-ANOVA (R^2^ = 0.56, Q^2^ = 0.45, CV-ANOVA: *p* < 0.001). The score plot showed a within-group separation based on pain intensity in FM. The loading plot showed four proteoforms that were associated with mild pain intensity (VAS < 45 mm), seven proteoforms that were associated with moderate pain intensity (VAS 45–74 mm), and two proteoforms that were associated with severe pain intensity (VAS > 75 mm). The nine proteoforms that were associated with severe and moderate pain intensity were one upregulated proteoform of alpha-2-macroglobulin (spot number 4808), serotransferrin (spot number 7404), and downregulated proteoform of ceruloplasmin (spot number 3810), haptoglobin beta chain (spot number 5104), antithrombin-III (spot number 3207), hemopexin (spot number 6403), alpha-2-macroglobulin (spot number 5905), immunoglobulin J chain (spot number 1007), and clusterin (spot number 1107) (Fig. 3 and Table 3). Additionally, a correlation analysis of VAS and the significant proteoforms from the OPLS model of VAS was analyzed, which showed 10 of 13 proteoforms had a correlation with pain intensity in FM (Supplementary Figure S2).

**Table 3:** Pain intensity and associated plasma proteins in FM*.*

Spot number	Protein name	Accession number	VIPpred	p(corr)	FM median OD (interquartile range)	CON median OD (interquartile range)	Fold change FM versus CON
**Mild pain intensity (5–44)**							
6103	Fibrinogen beta chain	P02675	1.21	-0.67	465 (173)	449 (200)	1.04
8508	Fibrinogen alpha chain	P02671	1.15	-0.64	383 (266)	382 (343)	1.00
8402	Fibrinogen alpha chain	P02671	1.12	-0.62	275 (292)	245 (185)	1.12
6203	Beta-2-glycoprotein 1	P02749	1.06	-0.58	747 (290)	663 (251)	1.13
**Moderate pain intensity (45–74)**							
3810	Ceruloplasmin	P00450	1.31	+ 0.72	203 (169)	216 (174)	0.94
5104	Haptoglobin beta chain	P00738	1.18	+ 0.65	112 (394)	226 (349)	0.50
3207	Antithrombin-III	P01008	1.15	+ 0.64	669 (256)	687 (257)	0.97
4808	Alpha-2-macroglobulin	P01023	1.13	+ 0.63	392 (421)	320 (829)	1.22
7404	Serotransferrin	P02787	1.13	+ 0.63	350 (308)	310 (291)	1.13
5905	Alpha-2-macroglobulin	P01023	1.07	+ 0.59	328 (541)	455 (388)	0.72
6403	Hemopexin	P02790	1.04	+ 0.57	1,047 (746)	1,198 (556)	0.87
**Severe pain intensity (75–100)**							
1007	Immunoglobulin J chain	P01591	1.13	+ 0.62	405 (489)	526 (415)	0.77
1107	Clusterin	P10909	1.01	+ 0.56	1,546 (645)	1,650 (832)	0.94

Plasma proteins with a VIP > 1, associated with pain intensity in the FM group. Spot numbers refers to equal numbers in Fig. 3 and marked proteins spots in supplementary Figure S1 and Table S1. Accession numbers are according to the protein data base UniProt (www.uniprot.org). Variable influence on projections (VIP) indicates the importance of the regressor, the higher value, the more important for the model. A VIP > 1 is considered as a significant protein. p(corr) is shown and is the correlation coefficient of each variable (protein) in the model. A positive p(corr) for a variable indicates a positive multivariate correlation with the Y-variable (pain intensity), and a negative p(corr), indicates a negative multivariate correlation with pain intensity in FM. The median quantified intensity (optical density, OD) from each group is shown in the table as a comparison. As descriptive values, protein fold change in FM compared to CON is reported. Please note that fold change is a univariate measure and do not necessarily correspond to p(corr); i.e., a positive fold change (FM > CON) is not automatically equal to a positive p(corr). The CON group is not included in the OPLS analysis.

#### Pathway analysis of proteins associated with pain intensity in FM

**Figure 4:**
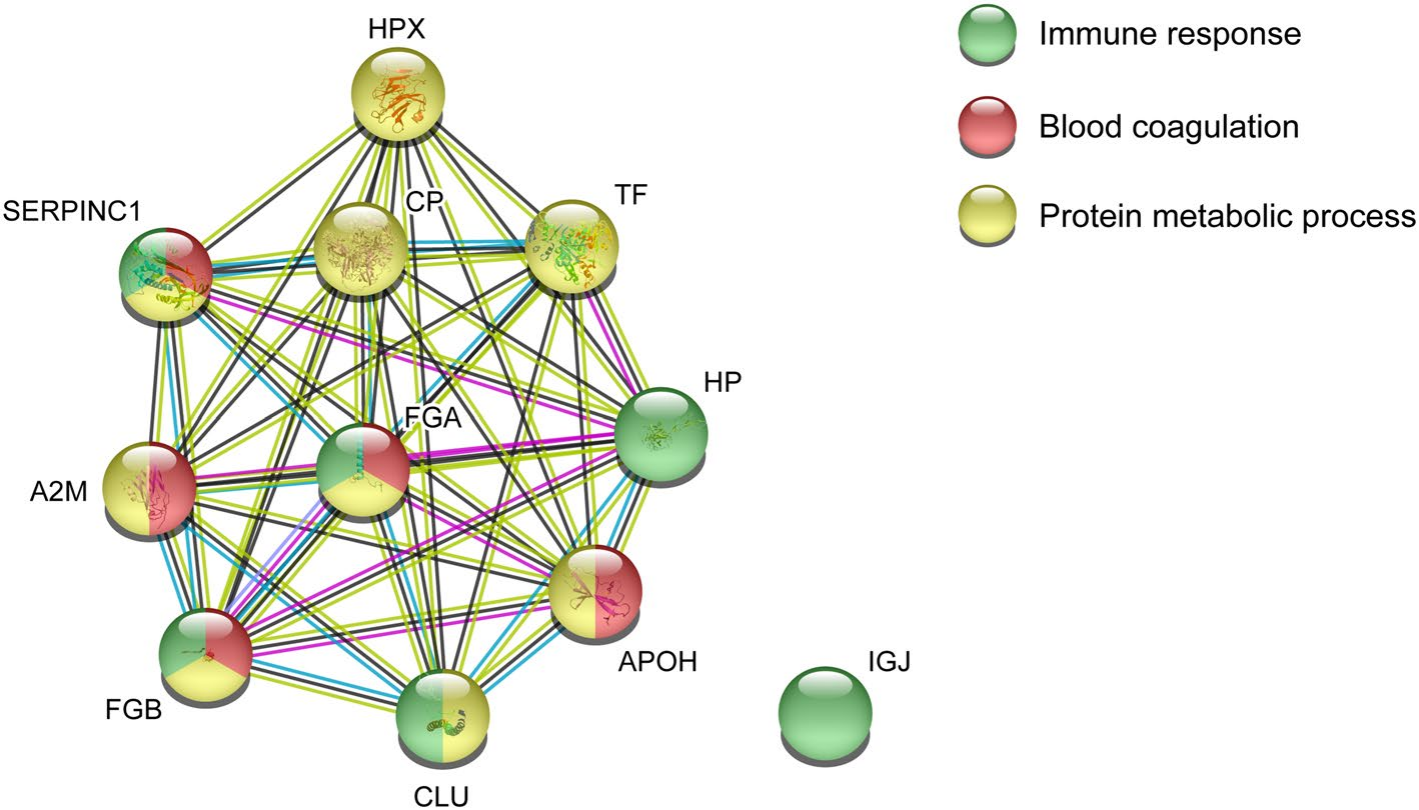
Pathway analysis of pain intensity in FM. Protein–protein network analysis of plasma proteins correlated with pain intensity in FM. The STRING version 11 was used to create the network analysis (https://string-db.org/). *A2M* Alpha-2-macroglobulin, *HP* Haptoglobin, *IGJ* Immunoglobulin J chain, *FGB* Fibrinogen beta chain, *FGA* Fibrinogen alpha chain, *TF* Serotransferrin, *APOH* Beta-2-glycoprotein 1, *CP* Ceruloplasmin, *CLU* Clusterin, *SERPINC1* Antithrombin-III, *HPX* Hemopexin.

Based on the result of the OPLS model of pain intensity in FM, a functional protein network analysis was created using STRING analysis (Fig. 4). Eleven proteins were included in the identified network, forming a large cluster. Fibrinogen alpha chain interacted with almost all proteins except for Immunoglobulin J that lacked any functional interaction to the investigated proteins in the network. In the enriched network, several biological processes were identified, i.e., immune response (FDR = 0.00098), blood coagulation (FDR = 1.43E−05), and protein metabolic process (FDR = 0.00053). Most of the proteins were involved in metabolic and immunity processes.

### Pressure pain thresholds (PPT) in FM

**Figure 5:**
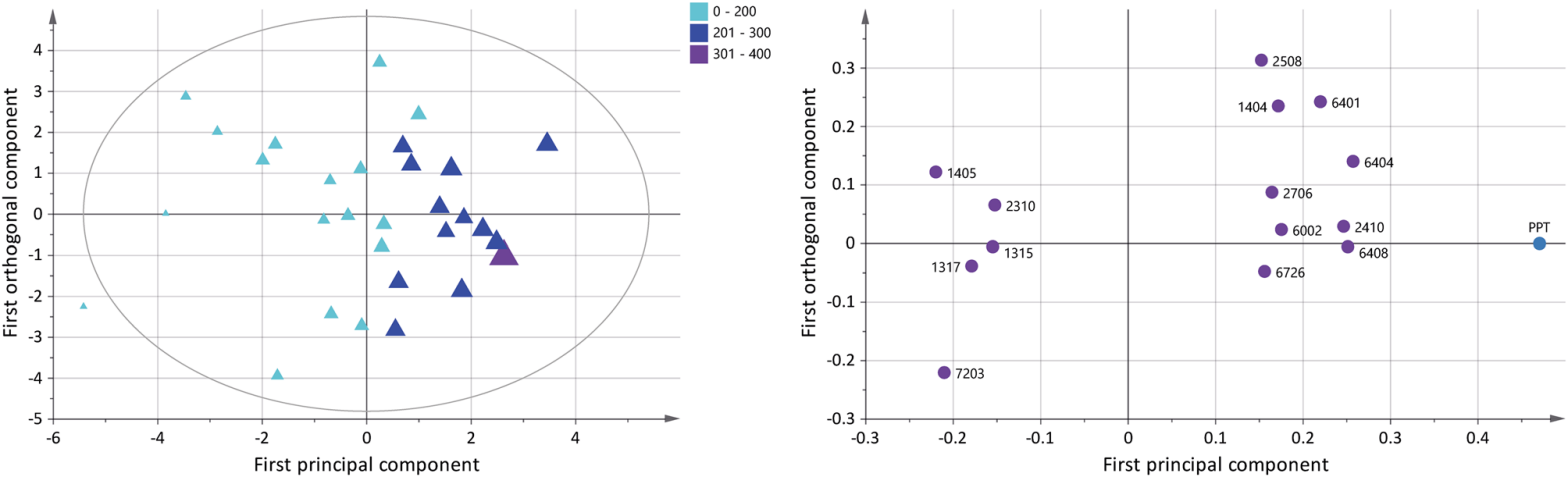
Pressure pain thresholds (PPT) and associated plasma proteins in FM. Score plot (left) shows a within-group separation in the FM group based on measured PPT. The larger the triangles, the higher the score. The loading plot (right) shows 14 proteoforms correlated with PPT (VIP > 1); five out of 11 proteoforms were associated with a very low pain sensitivity score (< 200 kPa). Numbers in the loading plot equals the spot numbers in Table 4 and supplementary Figure S1 and Table S1. The loading and score plots were created in SIMCA P + (version 15).

Ten unique proteins expressed as 14 proteoforms were multivariate correlated with PPT in FM according to the significant OPLS model (Fig. 5). The significant model had one predictive and one orthogonal component and showed a good explained variation, predictivity, and significant CV-ANOVA (R^2^ = 0.83, Q^2^ = 0.52, CV-ANOVA: *p* < 0.001). A within-group separation in the FM group is seen based on measured PPT and is visualized in the score plot. The loading plot showed five out of 14 proteoforms were associated with very low PPT (< 200 kPa), while nine proteoforms were associated with slightly higher PPT (201–300 kPa). The five proteoforms associated with very low PPT were upregulated proteoforms of angiotensinogen (spot number 1405), alpha-2-HS-glycoprotein (spot number 1317), fibrinogen beta chain (spot number 7203), and one upregulated and one downregulated proteoform of alpha-1-antitrypsin (spot number 1315 and 2310), respectively (Fig. 5 and Table 4). Additionally, a correlation analysis of PPT and the significant proteoforms from the OPLS model of PPT was analyzed, which showed six of 14 proteoforms had a correlation with PPT in FM (Supplementary Figure S3).

**Table 4:** Pressure pain thresholds (PPT) and associated plasma proteins in FM.

Spot number	Protein name	Accession number	VIPpred	p(corr)	FM median OD (interquartile range)	CON median OD (interquartile range)	Fold change FM versus CON
**PPT < 200 kPa**							
1405	Angiotensinogen	P01019	1.47	− 0.51	877 (796)	806 (606)	1.09
7203*	Fibrinogen beta chain	P02675	1.40	− 0.49	2,726 (1,121)	1,712 (1,708)	1.59
1317	Alpha-2-HS-glycoprotein	P02765	1.20	− 0.42	1,812 (2,439)	1,153 (2,251)	1.57
1315	Alpha-1-antitrypsin	P01009	1.03	− 0.36	5,329 (2,037)	4,680 (1,740)	1.14
2310	Alpha-1-antitrypsin	P01009	1.02	− 0.35	23,970 (5,242)	24,682 (4,898)	0.97
**PPT 201–300 kPa**							
6404	Hemopexin	P02790	1.73	+ 0.60	1,027 (592)	1,091 (494)	0.94
6408	Ig alpha-2 chain C region	P01877	1.69	+ 0.59	1,543 (1,447)	1,957 (1,386)	0.79
2410*	Alpha-2-antiplasmin	P08697	1.66	+ 0.58	538 (648)	818 (1,446)	0.66
6401	Hemopexin	P02790	1.48	+ 0.51	1,916 (365)	1,854 (502)	1.03
6002	Ig kappa chain C region	P01834	1.18	+ 0.41	682 (767)	588 (815)	1.16
1404*	Kininogen-1	P01042	1.15	+ 0.40	339 (204)	468 (280)	0.72
2706	Alpha-1-antitrypsin	P01009	1.11	+ 0.39	12 (30)	10 (32)	1.22
6726*	Complement C3b alpha chain	P01024	1.05	+ 0.37	185 (226)	252 (116)	0.73
2508	Alpha-2-antiplasmin	P08697	1.03	+ 0.36	71 (108)	102 (136)	0.69

Plasma proteins with a VIP > 1 associated with PPT in FM. Spot numbers refers to equal numbers in Fig. 5 and marked proteins spots in supplementary Figure S1 and Table S1. Accession numbers are according to the protein data base UniProt (www.uniprot.org). Variable influence on projections (VIP) indicates the importance of the regressor, the higher value, the more important for the model. A VIP > 1 is considered as a significant protein. p(corr) is shown and is the correlation coefficient of each variable (protein) for the model. A positive p(corr) for a variable indicates a positive multivariate correlation with the Y-variable (PPT), and a negative p(corr), indicates a negative multivariate correlation with PPT in FM. The median quantified intensity (optical density, OD) from each group is shown in the table as a comparison. As descriptive values, protein fold change in FM compared to CON is reported. Please note that fold change is a univariate measure and do not necessarily correspond to p(corr); i.e., a positive fold change (FM > CON) is not automatically equal to a positive p(corr). The CON group is not included in the OPLS analysis.

*Indicates shared proteoform with the model of group differences in FM and CON.

#### Pathway analysis of proteins related to PPT in FM

**Figure 6:**
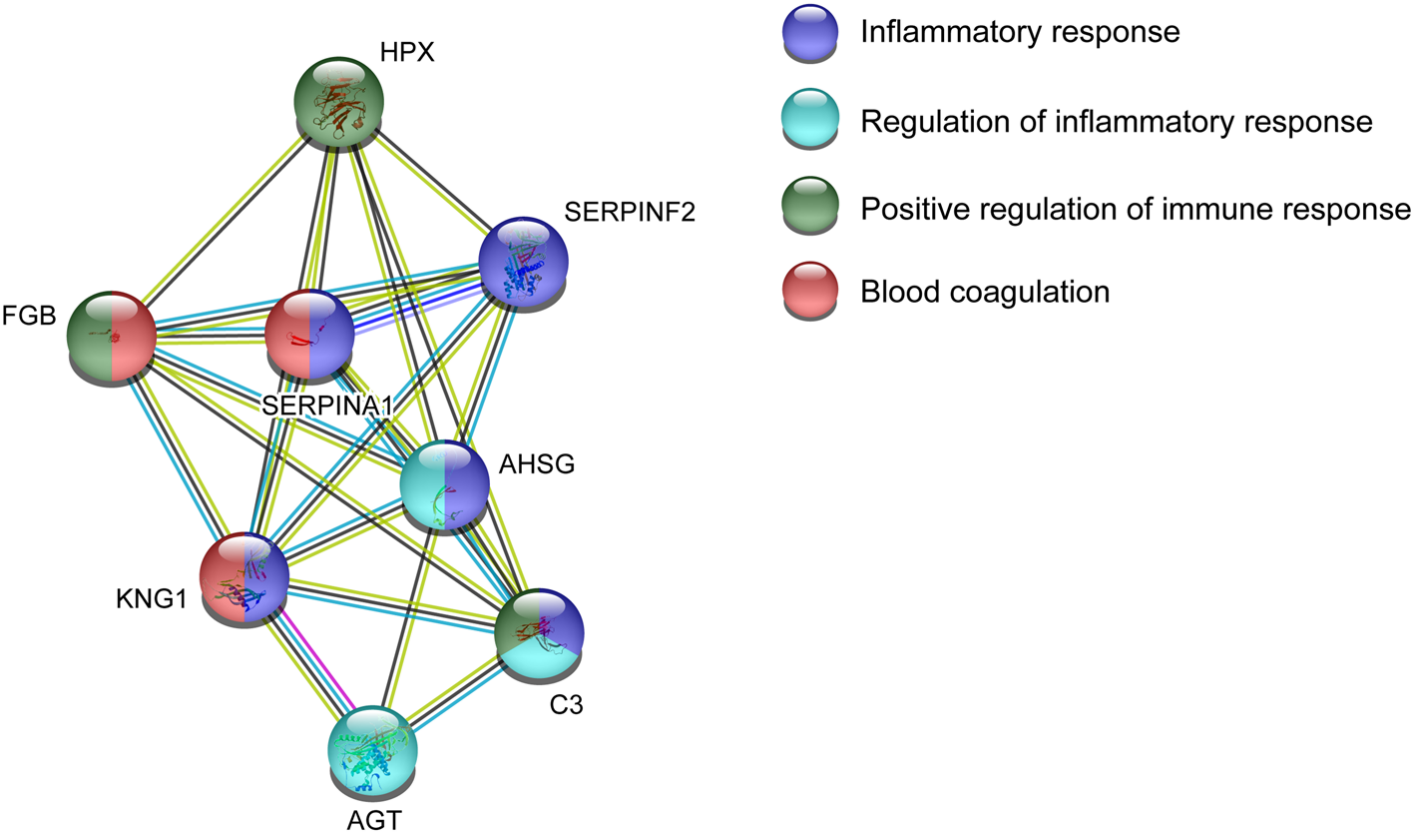
Pathway analysis of pressure pain thresholds (PPT) in FM. Functional protein network analysis of significant plasma proteins associated with PPT in FM. The STRING version 11 was used to create the network analysis (https://string-db.org/). *C3* Complement C3, *FGB* Fibrinogen beta chain, *SERPINA1* Alpha-1-antitrypsin, *SERPINF2* Alpha-2-antiplasmin, *KNG1* Kininogen-1, *AGT* Angiotensinogen, *AHSG* Alpha-2-HS-glycoprotein, *HPX* Hemopexin.

The significant proteins from the OPLS model of PPT in FM were analyzed to explore the functional protein network using STRING analysis (Fig. 6). Eight of ten proteins were included in the identified network (Ig alpha-2 chain C region and Ig kappa chain C region was not detected) and showed a relatively large cluster with several protein interactions. Kininogen-1 showed interaction with all proteins included in the network, especially with alpha-1-antitrypsin and angiotensinogen. The enriched protein network identified biological processes that were involved in, i.e., inflammatory response (FDR = 1.93E−05), regulation of inflammatory response (FDR = 0.0023), positive regulation of immune response (FDR = 0.0076), and blood coagulation (FDR = 0.0017). Most of the proteins associated with PPT in FM belonged to inflammatory response.

### Psychological distress (HADS total) in FM

**Figure 7:**
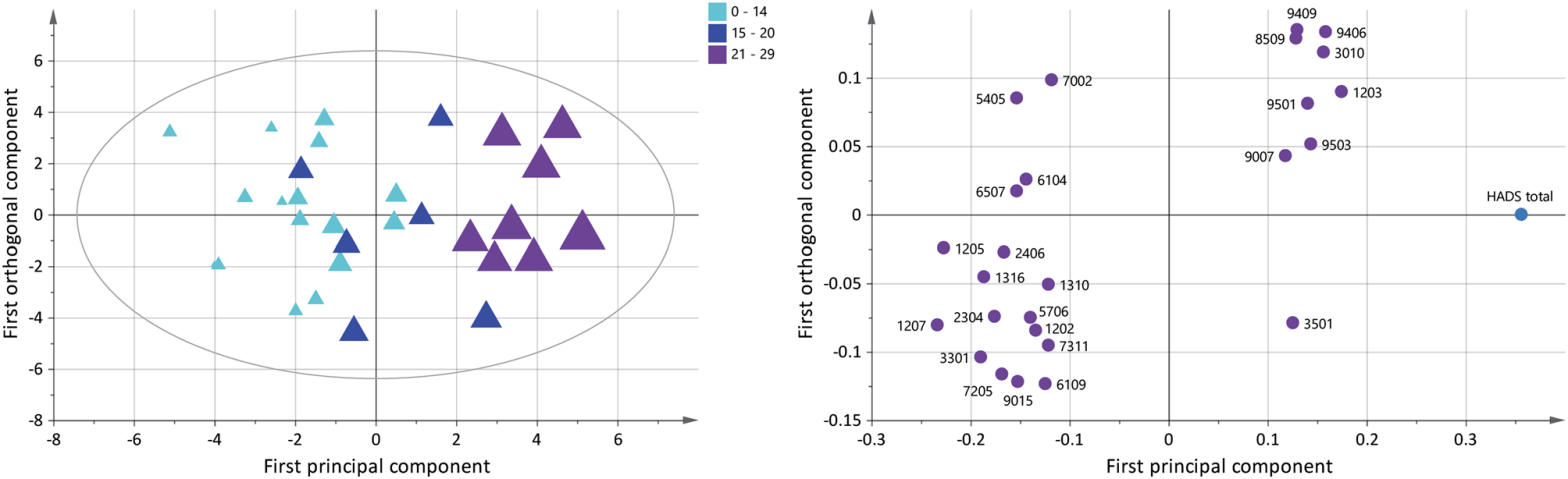
Psychological distress and associated plasma proteins in FM. Score plot (left) shows a within-group separation among the FM group based on HADS total score. The larger the triangles, the higher the score. The loading plot (right) shows 26 proteoforms correlated with HADS total (VIP > 1); eight out of 26 proteoforms were associated with moderate psychological distress (HADS total > 21). Numbers in the loading plot equals the spot numbers in Table 5 and supplementary Figure S1 and Table S1. The loading and score plots were created in SIMCA P + (version 15).

To assess plasma protein correlations to psychological distress in FM, an OPLS model was applied with the total score of HADS as dependent variable. The obtained significant model had one predictive and one orthogonal component with high explained variation, predictivity, and significant CV-ANOVA (R^2^ = 0.87, Q^2^ = 0.47, CV-ANOVA: *p* < 0.01). The score plot shows a within-group separation among FM based on psychological distress (Fig. 7). The loading plot shows 17 unique proteins, expressed as 26 proteoforms that were multivariate correlated with psychological distress in the FM group (Fig. 7). Seventeen proteoforms were associated with normal psychological distress (HADS < 14). One proteoform was associated with mild psychological distress (HADS = 15–20), and eight proteoforms were associated with moderate psychological distress (HADS > 21). The eight proteoforms associated with moderate psychological distress in FM were downregulated proteoforms of alpha-2-HS-glycoprotein (spot number 1203), apolipoprotein A-I (spot number 3010), Ig kappa chain C region (spot number 9007), fibrinogen beta chain (spot number 9409), two proteoforms of fibrinogen alpha chain (spot number 9503 and 9501), and one upregulated proteoform of fibrinogen alpha chain (spot number 8509) (Fig. 7 and Table 5). Additionally, a correlation analysis of HADS and the significant proteoforms from the OPLS model of HADS was analyzed, which showed 17 of 26 proteoforms had a correlation with psychological distress in FM (Supplementary Figure S4).

**Table 5:** Plasma proteins associated with psychological distress in FM.

Spot number	Protein name	Accession number	VIPpred	p(corr)	FM median OD (interquartile range)	CON median OD (interquartile range)	Fold change FM versus CON
**Normal distress (HADS < 14)**							
1207	Alpha-2-HS-glycoprotein	P02765	2.02	− 0.70	308 (847)	579 (998)	0.53
1205	Alpha-2-HS-glycoprotein	P02765	1.96	− 0.69	1,225 (1,883)	1,041 (1,830)	1.18
3301	Vitamin D-binding protein	P02774	1.64	− 0.57	2,511 (2,182)	2,787 (2,446)	0.90
1316	Alpha-2-HS-glycoprotein	P02765	1.61	− 0.56	153 (429)	216 (274)	0.71
2304	Vitamin D-binding protein	P02774	1.52	− 0.53	887 (705)	1,247 (854)	0.71
7205	Fibrinogen beta chain	P02675	1.46	− 0.51	777 (353)	847 (278)	0.92
2406	Alpha-2-antiplasmin	P08697	1.44	− 0.50	1,054 (763)	1,114 (1,065)	0.95
5405	Hemopexin	P02790	1.33	− 0.46	2,014 (680)	1,868 (765)	1.08
6507	C4b-binding protein alpha chain	P04003	1.32	− 0.46	381 (182)	375 (277)	1.02
9015	Serum amyloid A-4 protein	P35542	1.31	− 0.46	628 (869)	487 (620)	1.29
6104	Ficolin-3	O75636	1.24	− 0.43	425 (196)	449 (168)	0.95
5706	Complement C1r subcomponent	P00736	1.21	− 0.42	99 (84)	133 (118)	0.74
1202	Alpha-2-HS-glycoprotein	P02765	1.16	− 0.41	1,373 (1,023)	910 (1,409)	1.51
6109	Unidentified	Unknown	1.08	− 0.38	88 (94)	71 (107)	1.23
1310	Kininogen-1	P01042	1.05	− 0.37	199 (130)	226 (125)	0.88
7311	Unidentified	Unknown	1.05	− 0.37	203 (127)	220 (116)	0.92
7002	Haptoglobin	P00738	1.02	− 0.36	79 (210)	72 (226)	1.09
**Mild distress (HADS 15–20)**							
3501	Alpha-1B-glycoprotein	P04217	1.08	+ 0.38	2,822 (726)	3,151 (576)	0.90
**Moderate distress (HADS > 21)**							
1203	Alpha-2-HS-glycoprotein	P02765	1.51	+ 0.53	1,546 (1,218)	1,702 (1,363)	0.91
9406	Fibrinogen alpha chain	P02671	1.36	+ 0.48	1,505 (1,762)	1,844 (1,177)	0.82
3010	Apolipoprotein A-I	P02647	1.35	+ 0.47	64,643 (16,886)	66,242 (17,870)	0.98
9503	Fibrinogen alpha chain	P02671	1.24	+ 0.43	2,844 (2,571)	3,283 (2,001)	0.87
9501	Fibrinogen alpha chain	P02671	1.21	+ 0.42	3,688 (2,915)	3,894 (2,478)	0.95
9409	Fibrinogen beta chain	P02675	1.12	+ 0.39	5,127 (2,617)	5,497 (2,201)	0.93
8509	Fibrinogen alpha chain	P02671	1.11	+ 0.39	2,802 (2,207)	2,736 (1,895)	1.02
9007**	Ig kappa chain C region	P01834	1.01	+ 0.35	1,167 (1,888)	1,883 (2,049)	0.62

Proteins with a VIP > 1 associated with psychological distress in FM. Spot numbers refers to equal numbers in Fig. 7 and marked proteins spots in supplementary Figure S1 and Table S1. Accession numbers are according to the protein data base UniProt (www.uniprot.org). Variable influence on projections (VIP) indicates the importance of the regressor, the higher value, the more important for the model. A VIP > 1 is considered as a significant protein. p(corr) is shown and is the correlation coefficient of each variable (protein) for the model. A positive p(corr) for a variable indicates a positive multivariate correlation with the Y-variable (HADS total), and a negative p(corr), indicates a negative multivariate correlation with psychological distress in FM. The median quantified intensity (optical density, OD) from each group is shown in the table as a comparison. As descriptive values, protein fold change in FM compared to CON is reported. Please note that fold change is a univariate measure and do not necessarily correspond to p(corr); i.e. a positive fold change (FM > CON) is not automatically equal to a positive p(corr). The CON group is not included in the OPLS analysis.

**Indicates shared proteoform with the model of group differences in FM and CON.

#### Pathway analysis psychological distress in FM

**Figure 8:**
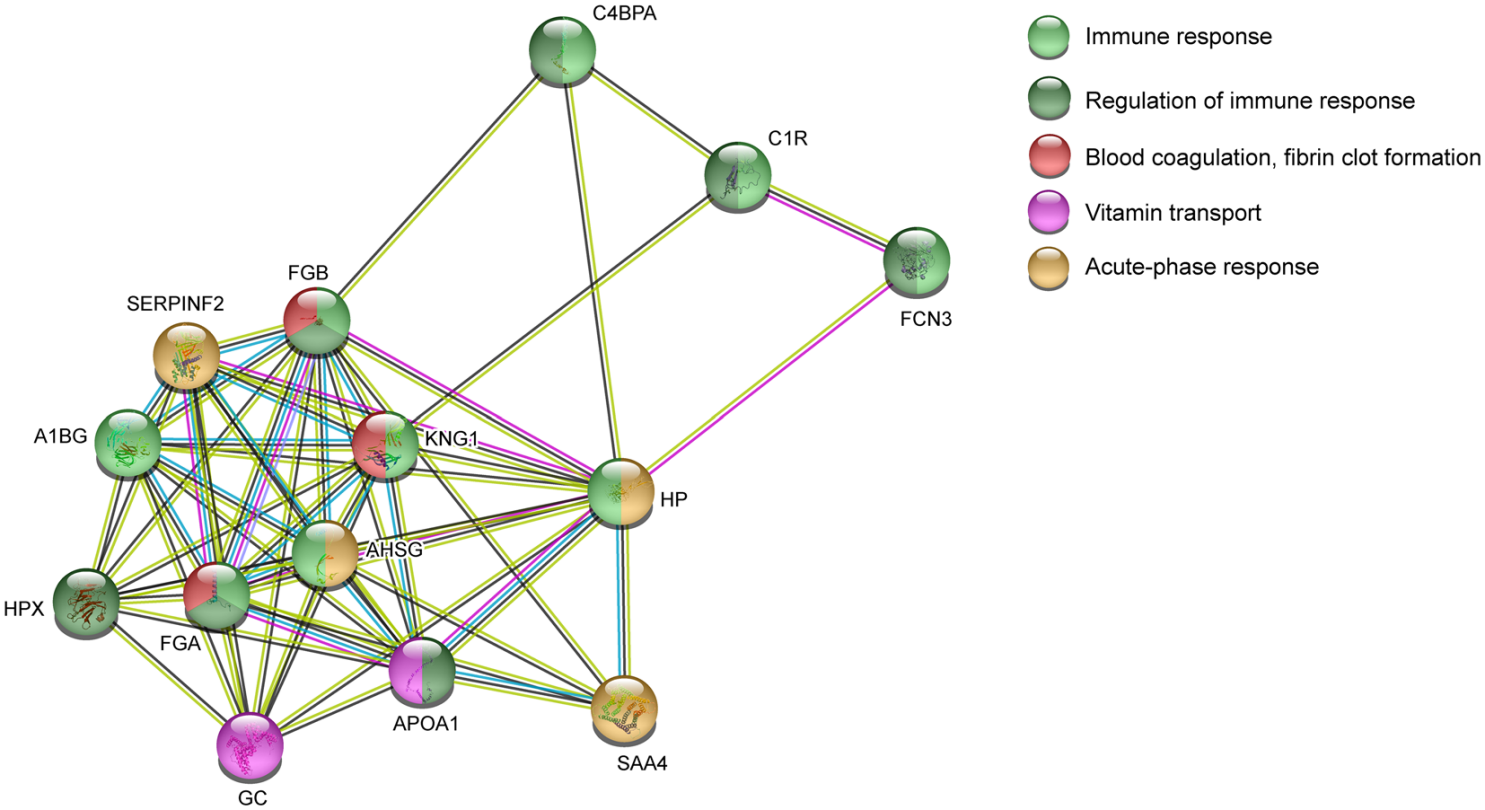
Pathway analysis of plasma proteins associated with psychological distress in FM. Functional protein network analysis of significant proteins associated with psychological distress in FM. The STRING version 11 was used to create the network analysis (https://string-db.org/). *FCN3*, Ficolin-3, *A1BG* Alpha-1B-glycoprotein, *HP* Haptoglobin, *FGB* Fibrinogen beta chain, *FGA* Fibrinogen alpha chain, *C4BPA* C4b-binding protein alpha chain, *SERPINF2* Alpha-2-antiplasmin, *KNG1* Kininogen-1, *APOA1* Apolipoprotein A-I, *AHSG* Alpha-2-HS-glycoprotein, *C1R* Complement C1r subcomponent, *HPX* Hemopexin, *SAA4* Serum amyloid A-4 protein, *GC* Vitamin D-binding protein.

The significant proteins from the OPLS model of psychological distress in FM were analyzed to explore functional protein network using STRING analysis. Fourteen of 17 proteins were included in the identified network (Ig kappa chain C region and the two unidentified proteins were not detected) and showed a large cluster with several protein interactions. Almost all proteins interacted with one another in the network, especially fibrinogen alpha chain, fibrinogen beta chain, and alpha-2-antiplasmin. The enriched protein network identified several biological processes, i.e., blood coagulation, fibrin clot formation (FDR = 3.40E−05), immune response (FDR = 8.92E−06), regulation of immune response (FDR = 2.88E−05), acute-phase response (FDR = 3.12E−06), and vitamin transport (FDR = 0.0029). Most of the proteins associated with the highest severity of psychological distress (i.e., moderate) belonged to blood coagulation, fibrin clot formation process (Fig. 8).

### Comparison between models, shared proteoforms, and accession numbers

**Figure 9:**
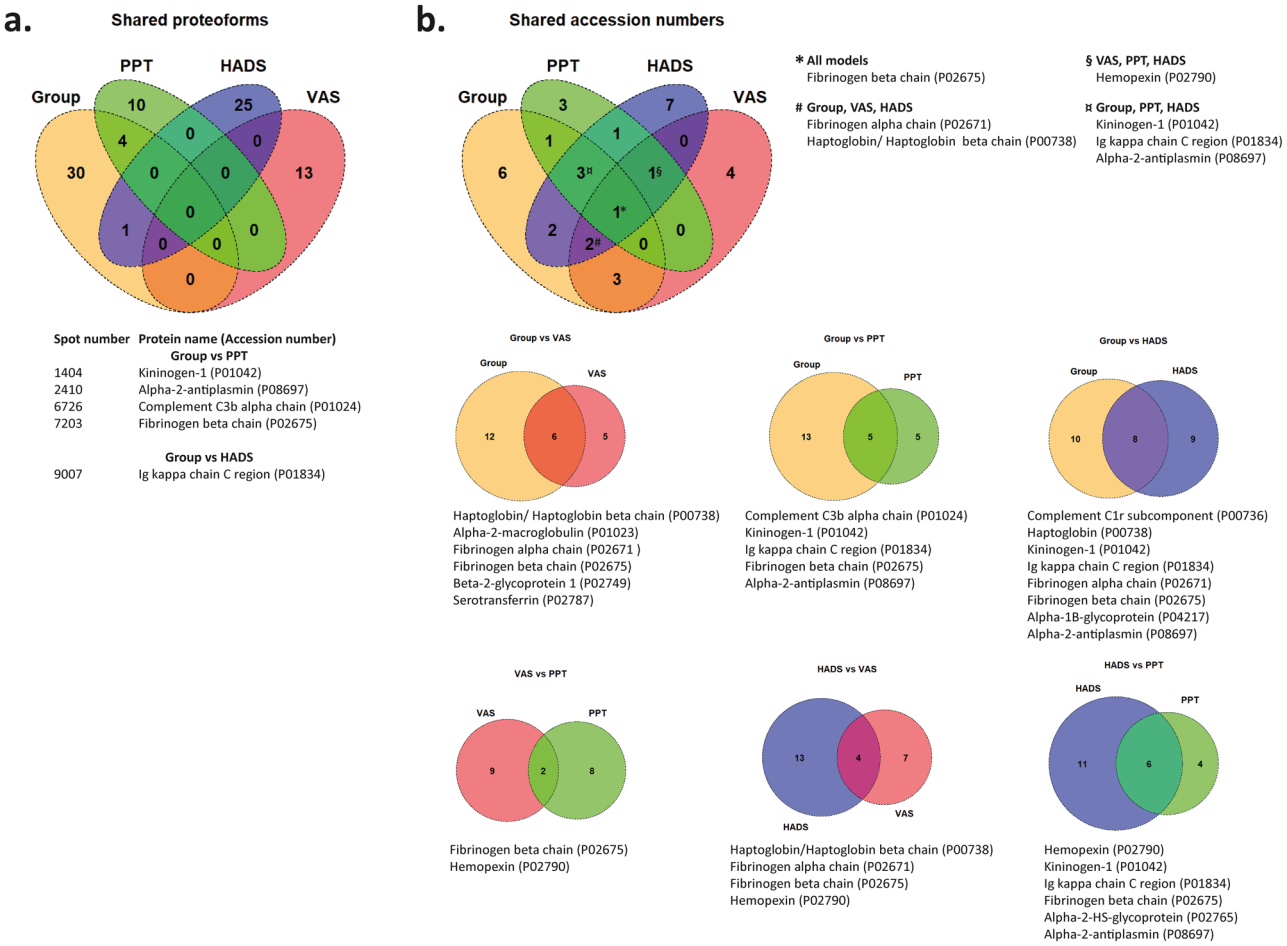
Venn diagram of shared proteoforms and proteins. (**a**) Venn diagram showing shared proteoforms between all models. *Group* group differences FM and CON, *PPT* PPT in FM, *HADS* Psychological distress in FM, *VAS* pain intensity in FM. Numbers prior protein name refer to unique spot numbers, while UniProt accession number for each protein is indicated in parenthesis. (**b**) Upper Venn diagram shows shared proteins between all four MVDA models. Venn diagrams below shows the comparison between two models individually, displaying the total number of shared proteins between respective models. UniProt accession number is indicated in parenthesis. The Venn diagrams were created in R using the Venndiagram package^73^.

To illustrate shared proteoforms and proteins between the above-presented models Venn diagrams were created. Four proteoforms; kininogen-1 (spot number 1404), alpha-2-antiplasmin (spot number 2410), complement C3b alpha chain (spot number 6726), and fibrinogen beta chain (7203) were significantly altered and shared between the models of group differences in FM vs. CON and PPT in FM (Fig. 9a, these proteoforms are marked in the respective table with *). One proteoform of Ig kappa chain C region (spot number 9007) was shared between the models of group differences in FM vs. CON and HADS in FM (Fig. 9a, this proteoform is marked in the respective table with **).

When comparing protein accession numbers between the models without taking proteoforms into account, several models shared the same type of protein, but not the same proteoforms (i.e., as indicated by spot number), e.g., fibrinogen beta chain was present in all models and hemopexin was present in the models of pain intensity, PPT, and psychological distress in FM (Fig. 9b).

### Adjusting for cofounding effects on significant proteoforms

The FM group had significantly higher BMI compared to CON. To evaluate if BMI had any confounding effect on significant proteoforms from each MVDA model, additional OPLS models were analyzed. An OPLS model was performed where only the FM group was included in the analysis. No significant model could be obtained (one principal component, R^2^ = 0.56, Q^2^ = 0.080, CV-ANOVA: *p* = 0.33). Further, when including both FM and CON in the analysis, the model was not significant (one principal component and one orthogonal component, R^2^ = 0.50, Q^2^ = −0.027, CV-ANOVA: *p* = 1). These two models confirm that BMI had no effect on the significant proteoforms identified in any of the MVDA models.

## Discussion

In this exploratory proteomic study, the major results were:

- Systemic differences between FM and CON were found in proteins involved in inflammatory, immunity, and metabolic processes.
- Pain intensity, sensitivity (PPT), and psychological distress in FM had associations with specific plasma proteins mainly involved in blood coagulation and processes of metabolism, inflammation, and immunity.

By analyzing the plasma proteome pattern of FM and CON using multivariate statistics and functional protein interaction network, significant differences in proteins involved in immunity, blood coagulation, metabolic, and inflammatory processes were found. These processes, among others, were also found in our previous study investigating the plasma proteome profile in women with CWP (mainly FM) compared to healthy controls^27^. Ramírez-Tejero et al*.*^31^ found upregulated levels of several proteins involved in acute phase response, the coagulation cascade, and the complement system, similar to the identified processes in this present study. Systemic differences in inflammatory proteins have also been found in farmers with musculoskeletal disorders^35^. Studies of other proteome profiles in FM, e.g., CSF, have additionally shown proteins involved in inflammatory response^11,25,34^. It is well known that there is interplay between the blood coagulation cascade and the complement system, and during inflammation, both these processes are highly active^36^. Therefore, it is not surprising that these processes are identified in the networks, both regarding the disease state (comparing FM and CON) but also when clinical pain characteristics are evaluated. Even though not the exact same proteins are found to be altered in this study compared to earlier studies, they are still involved in the same type of biological processes such as immunity, blood coagulation, metabolic, and inflammatory processes, which may overall point towards an inflammatory state among FM.

The most important proteoforms (with highest VIP and p(corr)) that were able to separate FM and CON, were downregulated proteoforms of Ig kappa chain C region, complement C-4B (fragment), fibrinogen alpha chain (fragment), and upregulated proteoforms of serotransferrin, alpha-2-antiplasmin, and haptoglobin (Table 2, Fig. 1). One proteoform of Ig kappa chain C region was downregulated, which partly confirms our previous results in the CWP cohort, where one of two proteoforms of Ig kappa chain C region were downregulated, however not the same proteoforms^27^. Further, several proteoforms of fibrinogen alpha chain (fragment) were downregulated. These proteoforms were not found significant in our previous study comparing CWP and CON^27^, but one (spot number 9106 with highest VIP and p(corr)) was found downregulated and associated with pain intensity in CWP^28^. Complement C-4B is part of the complement system and has its activity via the classical pathway. Two proteoforms were found significant, one upregulated (spot number 7101) and one downregulated (spot number 8101). The discrepancy in alteration of these proteoforms could be due to post-translational modification causing the p*I* to shift (which is seen in the location of the proteoforms in Supplementary Figure S1) or other modification which could affect the expressed levels; however, it might also be due to the disease state, which needs to be confirmed in more in-dept analysis. These two proteoforms were found upregulated in the model of CON and HADS total in our previous study^28^.

Regarding the upregulated proteins with highest VIP and p(corr), this study partly confirms our previous findings showing upregulated proteoforms of alpha-2-antiplasmin and haptoglobin. Though, in our previous study they were expressed as other proteoforms and as haptoglobin beta chain^27^. Serotransferrin was also confirmed in this study by two upregulated proteoforms; however, in the CWP cohort, another proteoform was found decreased^27^. Increased levels of specific serotransferrin proteoforms have been found in plasma from farmers with musculoskeletal disorders^35^ and in whole saliva from FM compared to controls^29^, which are in line with the findings in this study. Serotransferrin is known to be responsible for binding and transporting iron in the blood. Iron has been suggested to be involved in the pathophysiology behind FM^37^ via its acting as a cofactor in the production of the neurotransmitters dopamine and serotonin. Further, haptoglobin was also among the proteoforms with the highest VIP and p(corr). Haptoglobin is primarily produced in the liver and is a main transport of hemoglobin in the circulation. It is also classified as an acute-phase protein, due to its substantial increase during inflammation. It has been proposed that IL-6, among other cytokines released by macrophages and monocytes, affects hepatic cells to produce and, as a result, increase the haptoglobin concentration in the circulation^38,39^. In this cohort, we previously found increased plasma levels of the proinflammatory cytokines IL-6, IL-2, TNF-α, IP-10, and eotaxin^40^, which also have been seen in other studies of FM^11,41–43^. It is tempting to speculate that the increase in IL-6 specifically could explain the increase of two of the proteoforms of haptoglobin seen in FM in this cohort. However, it does not explain the decrease of another proteoform, which is the C-terminal part of haptoglobin (haptoglobin beta chain) that was seen in the model of pain intensity in FM. Increased levels of haptoglobin have been found in other studies investigating FM/CWP^25,31^, neuropathic pain^21^, and farmers with musculoskeletal disorders^35^. Haptoglobin’s involvement in FM needs to be further evaluated in additional studies. Overall, this present study further confirms that there is a possible alteration in proteins closely involved in the immune system, inflammation, metabolic, and blood coagulation processes in FM.

As expected and intended, the pain intensity was significantly higher in FM compared to CON (Table 1). In the score plot of FM and pain intensity, a within-group separation could be seen, which is based on the divided pain intensity categories (Fig. 3). By combining the score and loading plot, several proteins were associated with respective pain intensity category. Overall, the proteins that were associated with pain intensity were proteins involved in three different processes of immune response, blood coagulation, and protein metabolic process. These results confirm parts of our previous analysis of plasma proteins associated with pain intensity in CWP^28^, where ceruloplasmin, haptoglobin, different forms of fibrinogen (gamma and alpha chain), and clusterin were found altered. Pain intensity has been correlated to proteins involved in metabolic, stress, contractile, and inflammatory processes in muscle biopsies of the trapezius from the same CWP cohort^44^. Further, metabolic proteins were found in muscle biopsies from females with trapezius myalgia^20^. Overall, a vast majority of proteins associated with pain intensity belonged to metabolic processes in these and this present study. It seems that there is a specific metabolic protein profile in FM/CWP that is associated with pain intensity, which could be detected not only in peripheral muscle tissue but also systemically in FM.

Clusterin is suggested to be involved in inflammation due to its involvement in complement activation, apoptosis, and its ability to interact with immune cells such as macrophages and dendritic cells^45^. Interestingly, the proteoform of clusterin (spot number 1107, Table 3) associated with severe pain intensity was also found to be decreased in the CWP cohort^28^. Decreased level of clusterin has been found in serum from patients with erosive hand osteoarthritis, and negatively correlated with pain^46^.

Ceruloplasmin is one of the major acute-phase proteins that increases during inflammation and is responsible for the copper ion transport in the blood. One downregulated proteoform of ceruloplasmin was associated with moderate pain intensity (spot number 3810, Table 3). Upregulated levels of ceruloplasmin, however, expressed as another proteoform, and associated with pain intensity in CWP, were found in our previous study^28^. These two different proteoforms of ceruloplasmin were among the proteins with the highest VIP and p(corr) in each regression model from the respective studies. Further, elevated levels of ceruloplasmin have previously been found in FM patients compared to controls^47^. We also found several altered proteoforms of ceruloplasmin in CWP compared to controls^27^. Even though there are different proteoforms and/or total protein that were differentially altered in these studies, it seems that ceruloplasmin is involved in FM/CWP, and especially associated with pain intensity, which is one of the main clinical facets of FM. The function and influence of the specific proteoforms and downregulation of ceruloplasmin in this study need to be further elucidated in future studies. However, we cannot neglect that this protein shows up in several studies and might be involved in the pathophysiology behind FM. Several different forms of fibrinogen (alpha and beta) were upregulated and associated with pain intensity (Table 3), as in the CWP cohort^28^. Increased levels of blood coagulation proteins, e.g., fibrinogen, thrombin, have earlier been found in patients with chronic fatigue syndrome and/or FM^48^. It was also one of the major proteins, together with haptoglobin, that was elevated in the FM studied by Ramírez-Tejero et al.^31^. Our results further confirm that a specific protein profile for pain intensity in FM exists and that there is an increased metabolic and immunity process and decreased inflammatory process since several of the significant proteins that were involved in inflammation were downregulated in FM.

PPT is a semi-objective measurement of pain sensitivity, and several studies have shown reduced PPT in chronic pain conditions, especially FM/CWP compared to controls^24,44,49,50^. In agreement with this, we found that PPT was significantly reduced in the FM group compared to CON (Table 1). When investigating the plasma proteins related to PPT in FM, a within-group separation of the most important proteins was seen. The network analysis showed a distinct inflammatory profile, where elevated proteoforms of angiotensinogen, alpha-2-HS-glycoprotein, fibrinogen beta chain, and alpha-1-antitrypsin were associated with very low PTT in FM (Table 4). To the best of our knowledge, this is the first study to investigate the correlation between PPT and plasma proteins in FM. However, decreased PPT has been correlated with specific muscle proteins involved in stress, metabolic processes, and inflammation, in women with CWP compared to healthy controls^44^. Further, from the same CWP cohort, PPT was found negatively correlated with a specific cytokine/chemokine profile in CWP and CON^24^. Proteoforms of angiotensinogen and alpha-1-antitrypsin in CSF have also been found altered in neuropathic pain patients compared to controls^21^. Our study further confirms that specific plasma proteins involved in immunity/inflammation are associated with reduced PPT in FM.

We found significantly higher HADS total scores in FM compared to CON (Table 1). A within-group separation in FM was seen based on reported levels of psychological distress (Fig. 7). The majority of proteins associated with psychological distress in FM were proteins involved in acute-phase response, coagulation, and immunity process (Fig. 8). Previously, we have investigated the plasma proteome and association with psychological distress in CWP^28^. There, we found hemopexin, complement factor B, and clusterin, among others, to be positively associated with increased psychological distress in CWP. Different proteoforms of hemopexin were found in this study compared to the CWP cohort, however all proteoforms were elevated. Further, elevated proteoforms of hemopexin have been found in farmers with musculoskeletal disorder^35^. In addition, several proteomic studies of major depressive disorder have been conducted, revealing proteins to be involved in similar processes presented in this study, mainly acute-phase, blood coagulation, and immunity^51–53^. These studies indicate that the immune system and its proteins might be affected by elevated depression. However, it is important to stress that none of the FM patients in this cohort were clinically classified with major depression and/or anxiety, and that could therefore be one reason for the different expression of proteoforms in the aforementioned studies. Even though we were not able to confirm the same results in this FM group, as in the CWP cohort^28^, the same type of processes (immunity and blood coagulation) were identified. This study further confirms that there is a specific plasma protein pattern in FM associated with psychological distress, which in several aspects is similar to those seen in other patient groups experiencing depression and/or anxiety.

Four proteoforms were shared in the models of group differences and PPT in FM, and one proteoform was shared in the models of group differences and HADS total in FM (Fig. 9a). The discriminating proteoforms between the FM and the control group were also associated with pain sensitivity or moderate psychological distress in FM. Different proteoforms and alterations of fibrinogen beta chain were found in all MVDA models, and hemopexin was shared between the three clinical models. One explanation to the shared proteins/proteoforms may be that the clinical variables (pain intensity, pain sensitivity, HADS total, BMI, and age) were significantly correlated and hence confounded each other. However, a PCA of these variables in FM was not significant, which does not support such an explanation. These re-occurring proteins might reflect processes that are of importance and involved in the clinical aspects of FM. It is evident that not only central mechanisms are involved in FM, but also that peripheral factors play an important role in the mechanisms underlying FM.

Even though plasma has several advantages and is routinely used in clinical settings to analyze, e.g., acute-phase proteins, few studies have focused on the plasma proteome in chronic pain conditions, specifically FM. Several limitations exist when analyzing the plasma proteome. Plasma is a challenging fluid to study since it contains a high dynamic range of proteins with different abundance^54^. Depending on the choice of analyzing technique and its sensitivity, several orders of magnitude of both high and low abundant proteins can be studied, but the full dynamic range of plasma proteins is still challenging to analyze using proteomics. To reduce complexity, fractionation could be used (either protein or peptide level). Another common method to reduce complexity is to remove the top abundant proteins in plasma. In this study, we have chosen to deplete two of them, albumin and immunoglobulin G, which might have eliminated potential protein candidates, but also visualized other masked proteins of interest at a similar molecular weight and/or p*I*. Two-dimensional gel electrophoresis is a useful technique not only to visualize a protein’s molecular weight and p*I* and, therefore, different proteoforms but also post-translational modifications. This has been shown in this study and previous ones to be of importance when comparing group differences and clinical characteristics among FM/CWP. For future considerations, retrieval of blood samples during a specific time point of the day must be standardized, which was not done in this study. In a recent plasma proteomic study, it has been shown that several plasma proteins have a diurnal variation, especially immunity proteins involved in the complement system and inflammatory proteins^55^. We have excluded patients with severe comorbidities/disorders since an important part of this project was an exercise intervention (reported elsewhere^56^). Nevertheless, as reported in Table 1, patients had higher psychological distress and BMI. Several studies have shown that an increased BMI could affect immunity proteins specifically complement system proteins^57,58^. Therefore, we cannot exclude that the whole proteomic profile is influenced by BMI, especially the inflammatory proteins seen in the different models. Hence, BMI has been shown to be associated with several clinical symptoms in FM^59,60^, and to investigate if BMI is involved in the pathophysiology of FM, further studies with a large number of subjects including FM with normal BMI and high BMI is necessary. In this study, the ACR 1990 classification criteria for FM was used, and not the newer criteria from 2010/2011 or 2016^8–10^. Some of the FM patients would perhaps not be diagnosed with FM if the newer criteria were used. In future studies, the ACR 1990 and 2010/2011 or 2016 criteria should be used in studies analyzing biomarkers in order to compare previous studies using ACR 1990 criteria and 2010/2011 criteria. Despite patients refraining from certain pharmacological treatments 48 h prior to examinations, the remaining drugs may have influenced the proteomic profile. Another major drawback of this study is that we have not used an additional method to confirm our results. Therefore, the results and interpretation from this study should be taken with precautions before any candidate biomarkers for FM could be presented.

To summarize, this study has identified activated metabolic, immunity, and inflammatory pathways in FM, where the presence of immunity process was common when comparing group differences and correlations with the clinical pain characteristics. Although the fold changes of several identified proteins were not high, this proteomic study shows the importance of studying a panel of multiple proteins instead of single proteins suggesting a group of protein candidates that might be investigated in future studies as biomarkers of pathophysiological mechanisms in FM. In this study, we cannot evaluate causality of protein changes and chronic pain, pain sensitivity, and psychological distress.

In conclusion, this study further shows that specific protein pain signatures are associated with different clinical pain characteristics such as pain intensity, pain sensitivity as well as psychological distress, symptoms that are part of the clinical picture in FM. The identified protein pain signatures, consisting of proteins involved in processes such as immunity, inflammation, metabolic, and blood coagulation cascades, might be useful steps to gain mechanistic insight into FM and in search of new future potential and target candidate markers.

## Material and methods

### Subjects

This study is part of a larger randomized controlled multicenter trial exploring the effects of resistance exercise and relaxation therapy in women with FM (Clinicaltrials.gov NCT01226784, 21 October 2010). For more details of the recruitment process, see previous publications^56,61^. The recruitment processes of all participants started in 2010, and data collection ended at all sites in 2013. Samples from 30 FM patients and 32 CON were available for analysis in this study. The ACR 1990 classification criteria were used to asses FM diagnosis^3^. This study was planned and initiated in 2009, before the 2010 criteria were recognized; therefore the ACR 1990 criteria were the best recognized at that time point. Further, the inclusion criteria for both FM patients and healthy CON were to be of working age (20–65 years). For the CON group, good health and no current pain should be present. Exclusion criteria for both groups were high blood pressure (> 160/90 mmHg), osteoarthritis in the hip or knee, other severe somatic or psychiatric disorders, primary causes of pain other than FM, high consumption of alcohol (audit > 6), participation in a rehabilitation program within the past year, regular resistance exercise or relaxation therapy twice a week or more, inability to understand or speak Swedish, and not being able to refrain from analgesics, NSAID, or hypnotics for 48 h prior to examinations. Severe somatic disorders were, for instance, such as rheumatic disorders (e.g., Rheumatoid arthritis, Systemic lupus erythematosus), neurological diseases (e.g., Multiple sclerosis, Amyotrophic lateral sclerosis), diabetes mellitus, and cardiovascular diseases. Psychiatric diseases excluded were, e.g., major depressive disorder, severe anxiety disorders, and psychosis.

The study was conducted at three centers in Sweden (Gothenburg, Linkoping, and Stockholm) and in accordance with the Helsinki Declaration and Good Clinical Practice. The Ethical Review Board at Karolinska Institutet in Stockholm approved the study (Dnr: 2010/1121-31/3). All participants received verbal and written information about the study, and after that, written informed consent was obtained from all the participants in this study. All participants were compensated economically for their participation in the study.

### Background data, questionnaires, and pain characteristics

All participants answered questionnaires, including general background data as age (years), weight (kg), height (m), and body mass index (BMI; kg/m^2^) was calculated. For FM patients, the number of tender points was recorded after manual palpation by an experienced examiner to verify the ACR 1990 diagnosis of FM, and the pain duration (years) was recorded.

For all participants, pain intensity (whole-body) present for the past seven days was recorded using VAS (0–100). The reported VAS score has in this study been divided into the following categories: mild (5–44), moderate (45–74), and severe (75–100) pain intensity^62,63^. To assess the severity of FM, the FIQ was used. It consists of 10 subscales that are disease- specific regarding disabilities and symptoms and range from 0 to 100. In this study, the FIQ total is presented and is the mean score of all ten subscales. A high score indicates a lower health status^64^.

The validated Swedish version of the HADS was used to assess anxiety and depression^65,66^. Each subscale of HADS, HADS-anxiety and HADS-depression, consist of seven items and scores from 0 to 21. In this study, the results from the two subscales were summarized and presented as the total score of HADS, hence, to indicate psychological distress^67^. The total HADS score was divided into three categories according to the original case definition based on the separate anxiety and depression scale^65^: normal psychological distress (< 14), mild psychological distress (15–20), and moderate psychological distress (> 21).

PPT were measured by a trained examiner, using an electronic pressure algometer (Somedic SenseLab AB, Sosdala, Sweden). The probe area was 1 cm^2^, and the algometer was held perpendicularly to the skin area and pressed with a speed of approximately 50 kPa/s. Before the start of the measurement, the participants were familiarized with the testing procedure and instructed to mark their PPT during the experiment by pressing on a button as soon as the sensation of “pressure” changed to “pain”. When pushing the stop button or when maximum pressure was reached (1,500 kPa), the examiner removed the algometer, and the pressure stopped immediately. Algometry was performed on eight of 18 tender points defined by ACR criteria from 1990^3^ over the following sites: bilaterally over the supraspinatus muscle (at origins above the scapula spine near the medial border), the lateral epicondyle (2-cm distal to the epicondyles), over the gluteus maximus (in upper outer quadrants of buttocks in anterior fold of muscle), and inside of the knee (at the medial fat pad proximal to the joint line). In this present study, a mean value of the PPT from all eight sites was used in the background data and MVDA. The PPT has been divided into three categories: PPT 0–200, PPT 201–300, and PPT 301–400. PPT 0–200 is classified as very low pain sensitivity in the statistical analysis.

### Blood collection

Venous blood samples were collected from each participant with a Vacutainer (BD Vacutainer Eclipse Blood Collection Needle, BD Diagnostics, Becton, Dickinson, and Company, New Jersey, USA) in 10 mL K_2_EDTA tubes (BD Vacutainer Plus Plastic K_2_EDTA Tubes, BD Diagnostics). The plasma was retrieved by centrifuging the blood samples for 30 min 1,500×*g* at RT immediately after collection. The plasma fraction was removed to a new tube before aliquoted and stored in − 86 °C until analysis.

### Proteomics

#### Plasma sample preparation

The details for sample preparation have been described in previous studies^19,27^. ProteoPrep Immunoaffinity Albumin and Immunoglobulin G depletion kit was used to deplete the top two abundant proteins in plasma, albumin, and immunoglobulin G (Sigma-Aldrich Co, St Louis, MO, USA). Forty µl of plasma from each subject was added to separate columns and depletion was achieved according to the manufacturer’s protocol. The samples were desalted with PD-10 columns (GE Healthcare, Little Chalfont, UK) and lyophilized. The lyophilized plasma proteins were resolved in urea sample buffer (8 M urea, 2% 3-((3-Cholamidopropyl) dimethylammonio)-1-propanesulfonate hydrate (CHAPS) (w/v), 0.3% Dithiothreitol (DTT), 0.5% Pharmalyte 3-10 (v/v), bromophenol blue), and total protein concentration was measured with 2-D Quant Kit (GE Healthcare) according to manufacturer’s instructions, as described in previous studies^19,27^.

### 2-DE

A random sequence generator (www.random.org) was used to randomize the samples before run. The procedure of 2-DE has been described in detail in previous studies^19,27^. Hundred µg of total protein was applied to 18 cm Immobiline DryStrip gel pH 3–10 non-linear (GE Healthcare) and run in the Ettan IPGphor 3 IEF System (GE Healthcare) for 38,000 Vhs overnight. Each IPG strips was stored at − 86 °C until run in second dimension. Prior run in second dimension, the IPG strips were equilibrated twice for 15 min in sodium dodecyl sulfate (SDS) equilibrium buffer (6 M urea, 4% SDS (w/v), 30.5% glycerol (w/v), 0.5 M Trizma-HCl), containing first 1% DTT (w/v) to reduce the disulfide bonds and secondly with 4.5% iodoacetamide (w/v) to alkylate sulfhydryl groups and bromophenol blue for tracing. Second dimension was performed vertically on Ettan DALTsix Electrophoresis Unit (Amersham Pharmacia Biotech, Uppsala, Sweden) according to the manufacturer’s protocol. The proteins from the IPG strips were transferred to pre-casted homogenous 12.5% acrylamide gels (GE Healthcare), and initially run for 1 h at 80 V, 10 mA/gel, followed by 600 V, 40 mA/gel until the bromophenol blue line reached the bottom of the gel (approx. 6 h). Immediately after run, each gel was placed in fixative solution (10% methanol (v/v), 7% acetic acid (v/v)) overnight. The gels were then fluorescently stained with SYPRO Ruby (Bio-Rad Laboratories, Hercules, CA, USA) overnight. Each gel was visualized using a charged coupled device camera (VersaDoc Imaging system 4,000 MP; BioRad Laboratories). 2-DE protein patterns were analyzed and quantified using the software PDQuest Advanced version 8.0.1 (Bio-Rad Laboratories). The amount of protein in a spot was assessed as background-corrected optical density, integrated over all pixels in the spot and expressed as optical density. Quantified and matched proteins needed to be present in at least 50% in one of the groups to be eligible for further multivariate data analysis, as described in previous studies^19,27^.

### In-gel digestion by trypsin and protein identification

Protein spots of interest were either identified by spot matching to previously identified plasma proteins^27,28^ or by in-gel digestion using mass spectrometry as follows. Briefly, the spots were excised from the gel and de-stained with 50% acetonitrile (ACN) in 25 mM ammonium bicarbonate (v/v), dehydrated with 100% ACN, and dried in SpeedVac before digested with trypsin (1:20 trypsin:protein, Promega Biotech AB, Nacka, Sweden) at 37 °C overnight. Supernatants were then transferred into a new tube, and a second extraction step in 5% trifluoroacetic acid in 50% ACN on shaking for 4 h was performed. The two supernatants were then pooled, dried in SpeedVac and stored at -20 °C until analyzed with mass spectrometry (MS). The tryptic peptides were analyzed using a nano liquid chromatography system (EASY-nLC, Thermo Scientific, Waltham, MA, United States) with a C18 column (100 mm × 75 µM, Agilent technologies, Santa Clara, CA, USA) coupled to an LTQ Orbitrap Velos Pro MS (Thermo Scientific). Mobile phase (buffer A) consisted of 0.1% formic acid (v/v) in dH_2_O and peptides were separated using a linear gradient increasing from 2 to 90% ACN including 0.1% formic acid (buffer B), for 30 min with a flow rate of 300 nL/min. MS1 scan was performed with a mass range of 380–2000 m/z in positive mode, and the resolution was set to 30,000. In MS2 scan (MS/MS), the top 20 peaks from the full scan were selected for fragmentation. Peaks with + 1 charge state were rejected, and dynamic exclusion was 30 s.

Acquired raw files were analyzed with the software MaxQuant (version 1.5.8.3) against the human Swissprot/UniProt database (downloaded April 2018), with the following parameters; enzyme digestion with trypsin, the maximum number of missed cleavages 2, minimum peptide length 7, maximum peptide mass was 4,600 Da, parent ion mass tolerance 4.5 ppm, fragment ion mass tolerance 0.5 Da. Fixed modification was carbamidomethylation of cysteine, and variable modifications were oxidation of methionine and N-terminal acetylation. Protein false discovery rate (FDR) was set to < 1%, and a minimum of 1 unique peptide was needed to be considered as identified.

### Statistics

Background data were tested for normality, and the non-parametric Mann–Whitney *U* test was used to calculate group differences with SPSS version 24 (International Business Machines Corporation (IBM), Armonk, New York, USA). A *p* value ≤ 0.05 was considered significant. Data are presented as median and interquartile range if not stated otherwise.

MVDA is often used when large omics data are handled, and in this study, we have investigated multivariate correlations between quantified plasma proteins and group belonging (FM and CON) with the statistical model OPLS-DA using SIMCA P + version 15 (Umetrics, Umeå, Sweden). The OPLS-DA is a supervised modeling method and is used to detect variables able to discriminate between FM and CON. Regressions were analyzed between the clinical variables pain intensity, PPT, psychological distress, and quantified plasma proteins in FM, respectively, using OPLS modeling. The respective clinical variable was used as a single y-variable, and x-variables were quantified plasma proteins in FM. OPLS modeling has the ability to detect systemic variation in x-variables that either correlates or not (orthogonal) to y-variables. Both the OPLS-DA and OPLS models were performed in two steps, as described in previous studies^25,27,28^. All variables were mean-centered and scaled to unit variance (UV scaling) and log-transformed if necessary. An initial principal component analysis (PCA) was created to investigate possible outliers in the data set. One of the control samples was marked as a critical outlier by Hotelling’s T^2^, and after reviewing the data, it was removed from further analysis, resulting in 31 controls. All quantified plasma proteins (regressors) were in the first step included in the analysis and significant proteins with a variable influence on projection (VIP) value ≥ 1 with a jacked-knifed 95% confidence interval not including zero were selected for a second new regression model and presented in the results. VIP is a value that indicates the relevance of each x-variables pooled over all dimensions and y-variables, e.g., which x-variables that best explain Y; in models with one predictive component and one or several orthogonal components VIPpredictive (VIPpred) is used. The higher the VIP value, the more important regressor for the model. Further, analysis of variance of cross-validated predictive residuals (CV-ANOVA) was used to validate the significant level of each model (*p* ≤ 0.05 was regarded as a significant model). R^2^ describes the *goodness of fit*, which is the fraction of the sum of squares of all the variables explained by a principal component. It is a measure of how well the model is explained by the data set^68^. A cross-validated Q^2^ describes the *goodness of prediction* of the model, which is the fraction of the total variation of the variables that can be predicted by a principal component. This has been well described in our previous studies^25,28^. Each x-variable (proteins) is expressed as loadings in a loading plot and has a value ranging from − 1 to + 1; note that this is a multivariate correlation coefficient. The larger the loading, the more impact on the model’s principal components. p(corr) is the x variable loadings scaled as a correlation coefficient and is ranging from − 1 to + 1. The closer to absolute p(corr) to 1, the stronger is the correlation. The score plot shows the observations (FM or CON), and by combining the score plot and loading plot, important variables can be coupled to specific observations or within-in group separations. The described parameters necessary to evaluate MVDA modeling (R^2^, Q^2^, number of principal/orthogonal components, CV-ANOVA, and *p* value) are presented as suggested guidelines by Wheelock and Wheelock^69^.

### Confounding factors on significant proteoforms and clinical variables using OPLS and PCA

To adjust for a potential confounding effect of BMI on significant proteoforms, additional OPLS models were analyzed. The first model included FM and all significant proteoforms from each clinical MVDA model as regressors and BMI as y-variable. The second OPLS model included FM and CON, the significant proteoforms from the OPLS-DA model (comparing group differences) as regressors and BMI as y-variable. The clinical variables were analyzed with a PCA, to investigate potential interactions between each variable.

### Correlation matrices clinical variables and significant proteoforms

Spearman’s correlation matrices were calculated including the clinical variables VAS, PPT, HADS and significant proteoforms from respective OPLS models, using the R package Corrplot (version 0.84)^70^ (Supplementary Figures S2–S4).

### Bioinformatics

Protein–protein association network analysis was created using the online data-base tool STRING version 11^71^. Protein accession numbers (UniProt) from significant proteins from each MVDA model was entered in the search engine (multiple proteins) with the following parameters: Organism *Homo sapiens*, the maximum number of interaction was query proteins only, interaction score was set to medium confidence (0.400), and an FDR of ≤ 0.01 was used when classifying the Biological Process (GO) of each protein. In the network, each protein is represented by a colored node, and protein–protein interaction and association is represented by an edge visualized as a colored lined (type of interaction). Known interactions used were from curated databases (turquoise) and experimentally determined (pink). Predicted interactions were gene neighborhood (green), gene fusion (red) and gene-co-occurrence (dark blue), and other interactions were text mining (yellow), coexpression (black), and protein homology (purple).

Venn diagrams and heatmap were created with the R software (version 3.6.1)/R studio (version 1.2.1335)^72^ with the packages VennDiagram (version 1.6.20)^73^ and heatmap.2 included in gplots (version 3.0.1.1)^74^.

## Supplementary information

Supplementary information
