# Supplementary material for: Significant correlation between plasma proteome profile and pain intensity, sensitivity, and psychological distress in women with fibromyalgia
Source: Sci Rep. 2020 Jul 27;10:12508. doi: 10.1038/s41598-020-69422-z (PMC7385654; doi:10.1038/s41598-020-69422-z)

Significant correlation between plasma proteome profile and pain intensity, sensitivity, and psychological distress in women with fibromyalgia

Karin Wåhlén, MSc[1]*, Malin Ernberg, DDS, PhD[2], Eva Kosek, MD, PhD[3], Kaisa Mannerkorpi, FT, PhD[4], Björn Gerdle, MD, PhD[1+], Bijar Ghafouri, PhD[1+]

[1]Pain and Rehabilitation Center, and Department of Health, Medicine and Caring Sciences, Linköping University, Linköping, Sweden.

[2]Department of Dental Medicine, Karolinska Institutet, and Scandinavian Center for Orofacial Neurosciences (SCON), SE-141 04 Huddinge, Sweden.

[3]Department of Clinical Neuroscience, Karolinska Institutet, SE-171 77, Stockholm, Sweden.

[4]Department of Health and Rehabilitation/Physiotherapy, Institute of Neuroscience and Physiology, Sahlgrenska Academy, Gothenburg University, Gothenburg, Sweden

[+]Shared last author.

*Corresponding author:

Karin Wåhlén,

Pain and Rehabilitation Center,

Department of Health, Medicine and Caring Sciences,

PAINOMICS® Laboratory

Linköping University

SE-581 85 Linköping, Sweden

Phone: +46 10 103 4155

Email: karin.wahlen@liu.se

Supplementary Table S1. Mass spectrometry data of identified proteins. Spot numbers correspond to marked protein spots on the two-dimensional gel in supplementary Figure S1 and numbers in respective table for each multivariate model. Part of the MS data have been published previously (see references in last column). For details of the methodology, see method section and previous published studies [1, 2, 3]. MW: molecular weight, p*I*: isoelectric point.

Spot number	Protein name	Accession number (UniProt)	Theoretical MW (kDa)/p*I*	Intensity	SCORE	MOWSE score	Number of peptides	Sequence coverage (%)	Published MS data
2706	Alpha-1-antitrypsin	P01009	46.7 / 5.4	2.16E+09	323	-	20	58.4	
1315				-	-	-	-	-	
2310				9.91E+09	323	-	25	60.8	[1]
3502	Alpha-1B-glycoprotein	P04217	54.3 / 5.4	1.62E+08	323	-	10	36.4	
3501				2.31E+09	323	-	14	45.3	
2406	Alpha-2-antiplasmin	P08697	54.6 / 5.9	-	-	-	-	-	[4]
2410				-	-	1.26E+04	12	28.9	
2508				-	-	2.40E+05	11	30.8	[1]
3403				-	-	-	-	-	[4]
4401				-	-	-	-	-	[4]
1202	Alpha-2-HS-glycoprotein	P02765	39.3 / 5.4	-	-	-	-	-	[4]
1203				-	-	-	-	-	[4]
1205				-	-	-	-	-	[4]
1207				-	-	-	-	-	[4]
1316				-	-	-	-	-	[4]
1317				-	-	2.18E+7	9	27.5	
4808	Alpha-2-macroglobulin	P01023	163.3 / 6.0	6.23E+08	323	-	31	26.1	[1]
4814				1.00E+08	323	-	31	23	
5903				1.20E+08	323	-	22	19.1	

5905	Alpha-2-macroglobulin	P01023	163.3 / 6.0	-	-	-	-	-	
1405	Angiotensinogen	P01019	53.2 / 5.9	9.88E+07	276	-	10	26.6	
3207	Antithrombin-III	P01008	52.6 / 6.3	2.03E+09	323	-	20	51.9	
3010	Apolipoprotein A-I	P02647	30.8 / 5.6	1.43E+10	323	-	26	70.0	
1004	Apolipoprotein C-III	P02656	10.9 / 5.2	3.21E+08	265	-	4	37.4	
6203	Beta-2-glycoprotein 1	P02749	38.3 / 8.3	1.16E+08	323	-	9	30.4	[1]
7206				7.46E+08	323	-	11	51.9	
6507	C4b-binding protein alpha chain	P04003	67.0 / 7.2	-	-	8.91E+6	13	22.8	
3810	Ceruloplasmin	P00450	122.2 / 5.4	-	-	-	-	-	[2, 4]
1107	Clusterin	P10909	52.5 / 5.9	3.82E+08	323	-	9	27.6	[1]
3728	Complement C1r subcomponent	P00736	80.1 / 5.8	-	-	2.98E+09	10	20.6	[1, 2]
5602				-	-	4.38	4	8.4	[1, 2]
5706				-	-	1.71E+12	19	30.6	[1, 2]
7403	Complement C3 beta chain	P01024	71.3 / 6.8	3.20E+07	267	-	18	40.9	
6726	Complement C3b alpha chain	P01024	103.9/5.2	1.63E+07	323	-	15	13	[1]
7101	Complement C4-B (fragment)	P0C0L5	33.1 / 6.4	-	-	5.23E+03	5	14.1	[1]
8101				-	-	3.19E+8	12	50.9	[1]
2113	Complement factor I light chain	P05156	27.6 / 6.2	-	-	1.58E+03	6	32.0	[1, 2]
8402	Fibrinogen alpha chain	P02671	94.9 / 5.7	-	-	-	-	-	[1, 4]
8413				-	-	-	-	-	[1, 4]
8508				-	-	-	-	-	[1, 4]
9503				-	-	-	-	-	[1, 4]
9406				-	-	-	-	-	[1, 4]
9501				4.39E+09	323	-	27	37.3	[1]
8509				-	-	-	-	-	[1, 4]
8510				1.79E+09	323	-	23	28.9	[1]
9105	Fibrinogen alpha chain fragment	P02671	91.3 / 5.7	-	-	-	-	-	
9106				4.07E+07	306	-	12	14.3	[1]
9110				-	-	-	-	-	
9108				-	-	-	-	-	

6103	Fibrinogen beta chain	P02675	55.9 / 8.5	1.17E+06	103	-	3	9.4	
7203	Fibrinogen beta chain	P02675	55.9 / 8.5	5.10E+08	323	-	19	48.9	
7205				1.94E+07	323	-	9	23.8	
9409				-	-	-	-	-	[1,4]
6104	Ficolin-3	O75636	32.9 / 6.2	-	-	3.12E+04	6	20.1	[1, 2]
6606	Gelsolin	P06396	85.7 / 5.9	-	-	1.64E+12	23	40.4	[2]
4107	Haptoglobin	P00738	45.2 / 6.1	6.71E+08	323	-	14	40.9	[1]
4103				6.62E+08	323	-	14	40.6	[1]
7002				5.22E+08	323	-	10	28.3	
5104	Haptoglobin beta chain	P00738	27.2 / 6.3	3.23E+06	101	-	2	6.9	
5405	Hemopexin	P02790	52.4 / 6.5	-	-	-	-	-	
6401				-	266	-	8	20	[2, 3]
6403				-	277	-	9	20	[3]
6404				-	49	-	1	2	[3]
6408	Ig alpha-2 chain C region	P01877	36.9 / 5.8	-	-	-	-	-	[2, 4]
9010	Ig kappa chain C region	P01834	11.6 / 5.6	-	-	-	-	-	[4]
9007				-	-	-	-	-	[4]
6002				-	-	1.14E+03	2	30.2	[1, 2]
1007	Immunoglobulin J chain	P01591	18.1 / 5.1	-	-	5.10E+03	7	50.3	
1404	Kininogen-1	P01042	71.9 / 6.3	4.95E+08	323	-	13	22.8	[1]
1310				2.59E+08	323	-	17	22.8	
8701	Plasminogen	P00747	90.6 / 7.0	-	-	9.09E+17	29	46.5	[1, 2]
8703				-	-	5.94E+12	22	30.6	[1, 2]
8706				-	-	-	-	-	
9701				-	-	-	-	-	[1, 2, 4]
7404	Serotransferrin	P02787	77.0 / 6.8	2.17E+08	323	-	38	50.7	
7619				-	-	-	-	-	[1, 4]
7621				-	-	-	-	-	[1, 4]
9015	Serum amyloid A-4 protein	P35542	14.7 / 9.1	1.52E+08	70	-	2	14.6	
6109	Unidentified	Unknown	-	-	-	-	-	-	

6205	Unidentified	Unknown	-	-	-	-	-	-	
7311	Unidentified	Unknown	-	-	-	-	-	-	
2304	Vitamin D-binding protein	P02774	52.9 / 5.4	-	-	1.22E+9	15	33.5	[2]
3301				-	-	-	-	-	

References

1. Wåhlén, K., B. Ghafouri, N. Ghafouri, and B. Gerdle, Plasma Protein Pattern Correlates With Pain Intensity and Psychological Distress in Women With Chronic Widespread Pain. Frontiers in Psychology, 2018. 9(2400).

2. Wåhlén, K., Olausson, P., Carlsson, A., Ghafouri, N., Gerdle, B., & Ghafouri, B. (2017). Systemic alterations in plasma proteins from women with chronic widespread pain compared to healthy controls: a proteomic study. J Pain Res, 10, 797-809. doi:10.2147/jpr.s128597.

3. Ghafouri, B., Carlsson, A., Holmberg, S., Thelin, A., & Tagesson, C. (2016). Biomarkers of systemic inflammation in farmers with musculoskeletal disorders; a plasma proteomic study. BMC Musculoskelet Disord, 17(1), 206. doi:10.1186/s12891-016-1059-y

4. Swiss Institute of Bioinformatics, SWISS 2D-PAGE, ExPASy Molecular Biology Server at https://world-2dpage.expasy.org/swiss-2dpage/

Supplementary Figure S1. Representative plasma proteome showing location of significant proteoforms. Protein spot numbers correspond to equal numbers in each multivariate data analysis model and MS data in Supplementary Table S1. For the original two-dimensional gel, uncropped and unadjusted contrast, see figure below. MW: molecular weight, p*I*: isoelectric point.

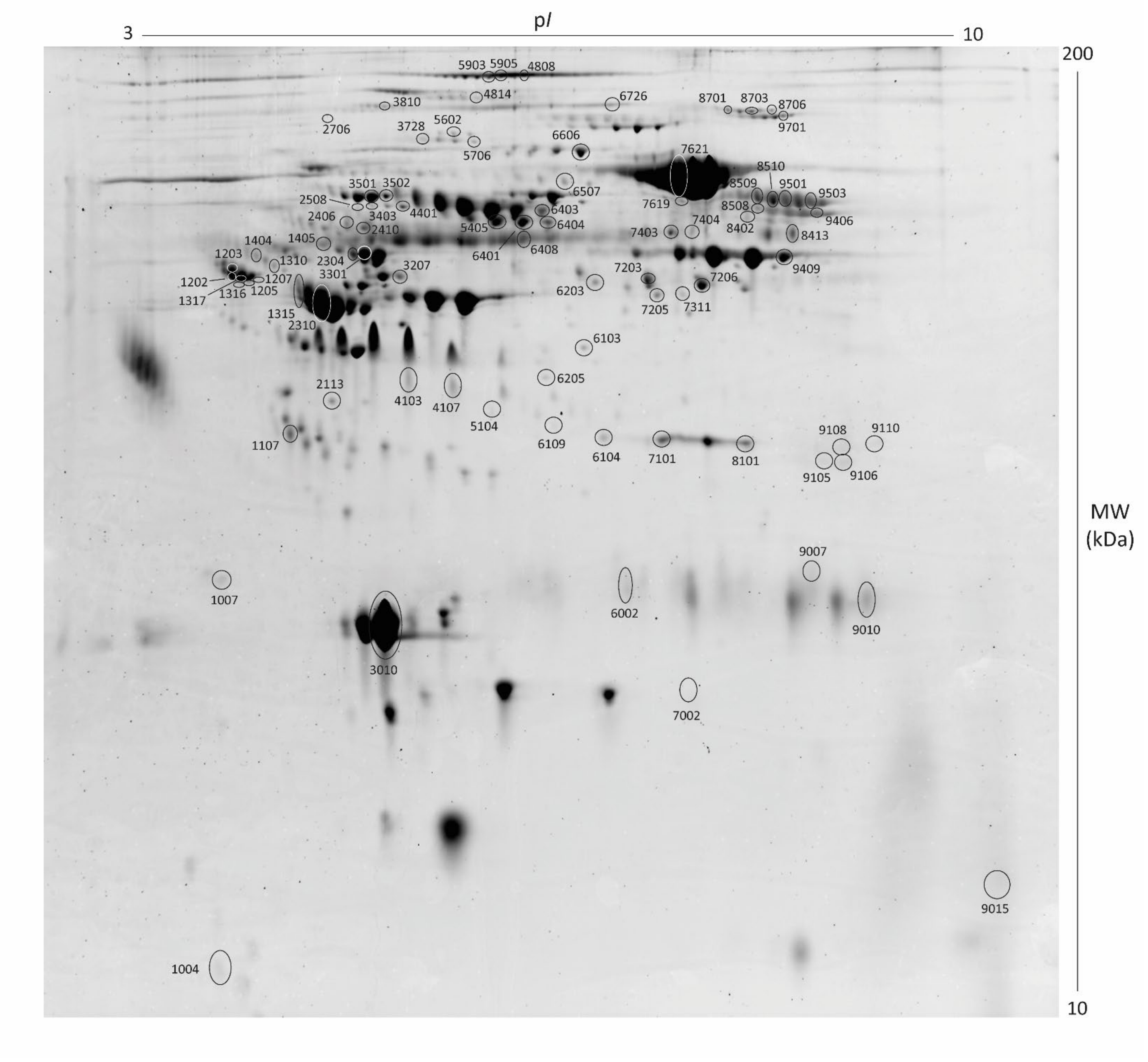

Original two-dimensional gel

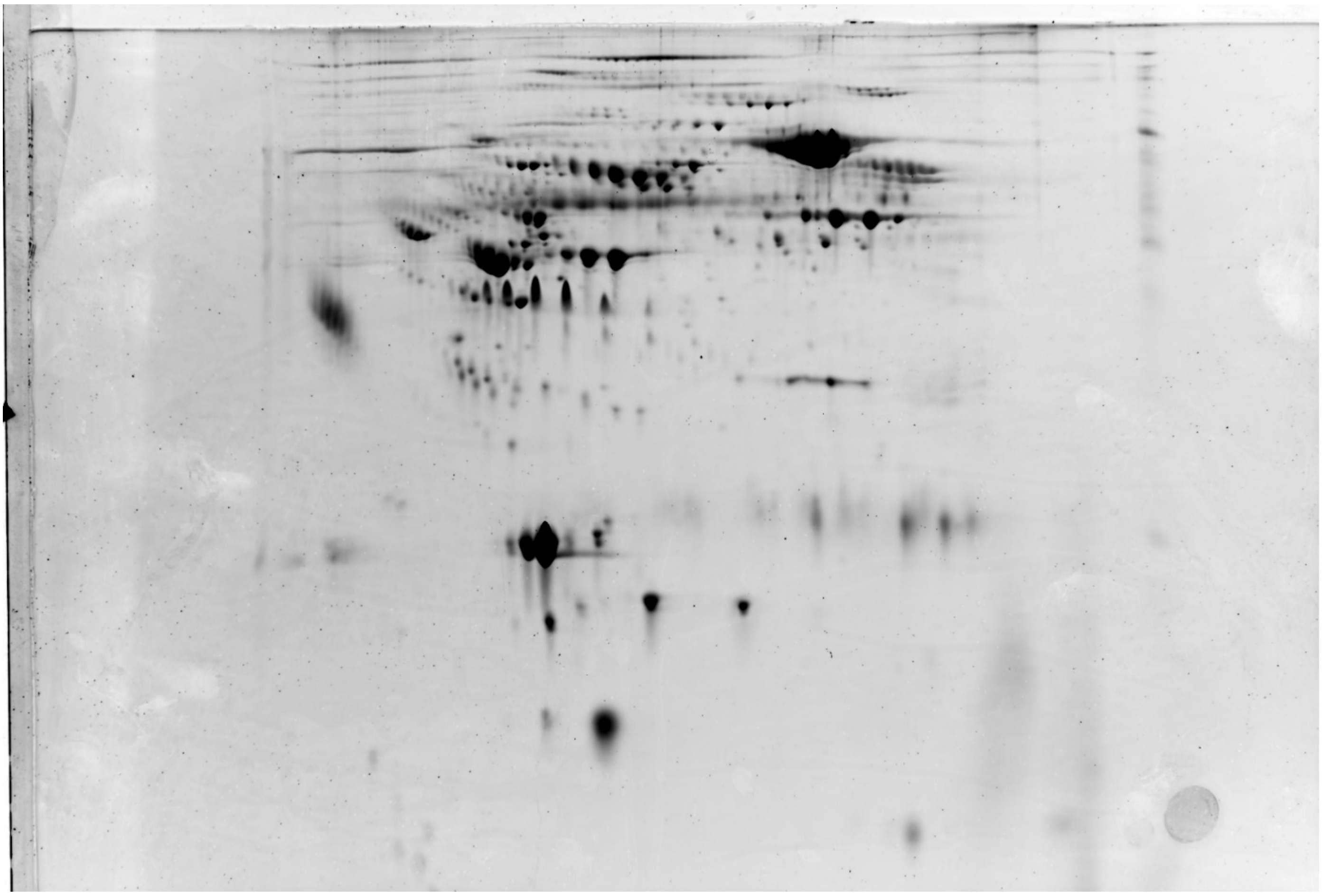

Supplementary Figure S2. **Correlation matrix of VAS and significant proteoforms in FM.** Spearman correlation matrix of VAS and significant proteoforms from the OPLS model of VAS in FM. Ten of the proteoforms had a significant correlation with VAS. The higher color intensity, the stronger correlation. Red = negative correlation, blue = positive correlation. Variable names are protein accession number and protein spot number, which are equal to presented number in Figure 3 and Table 3, and Supplementary Table S1 and Figure S1. * = p<0.05, ** = p<0.01, VAS = visual analogue scale.

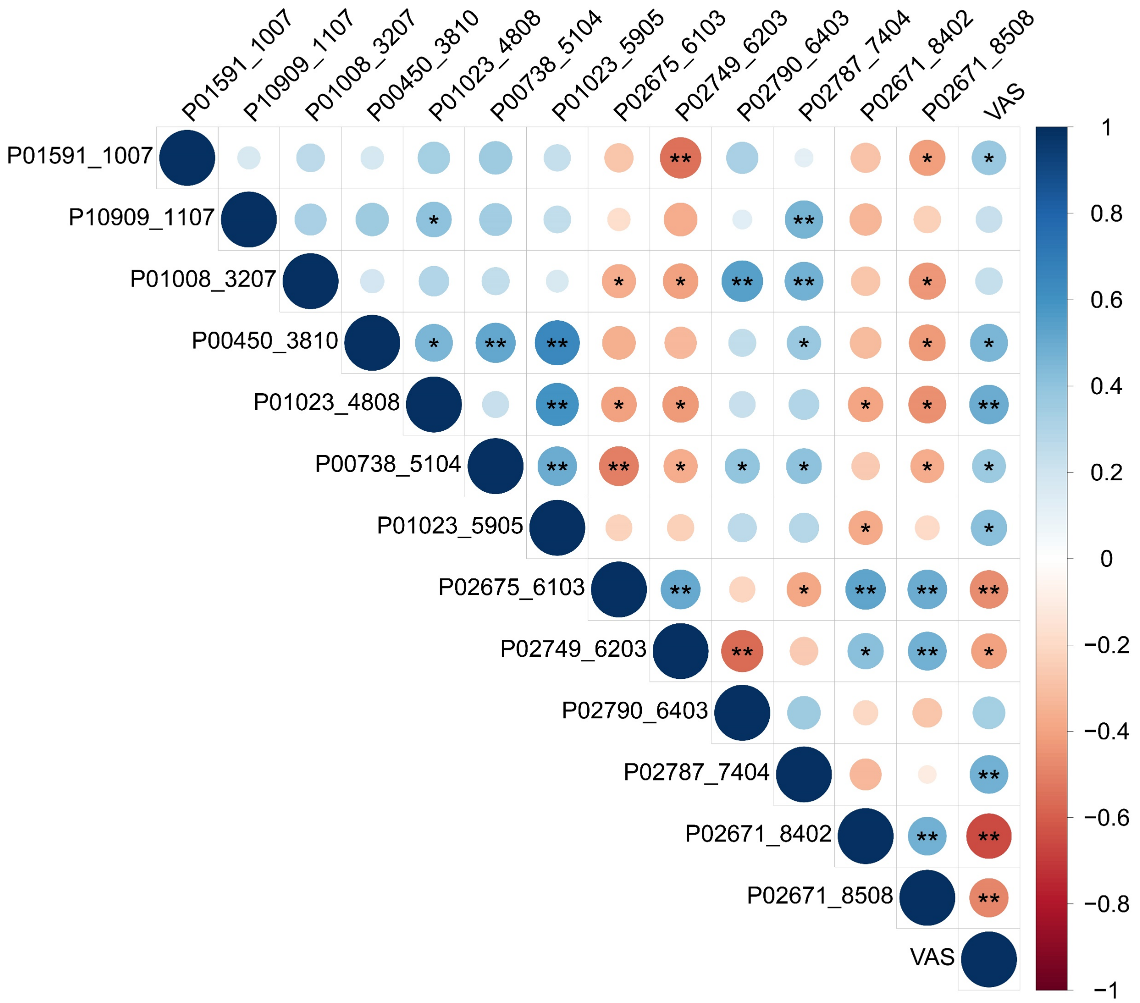

Supplementary Figure S3. Correlation matrix of PPT and significant proteoforms in FM. Spearman correlation matrix of PPT and significant proteoforms from the OPLS model of PPT in FM. Six of the proteoforms had a significant correlation with PPT in FM. The higher color intensity, the stronger correlation. Red = negative correlation, blue = positive correlation. Variable names are protein accession number and protein spot number, which are equal to presented number in Figure 4 and Table 4, and Supplementary Table S1 and Figure S1. * = p<0.05, ** = p<0.01, PPT = pressure pain thresholds.

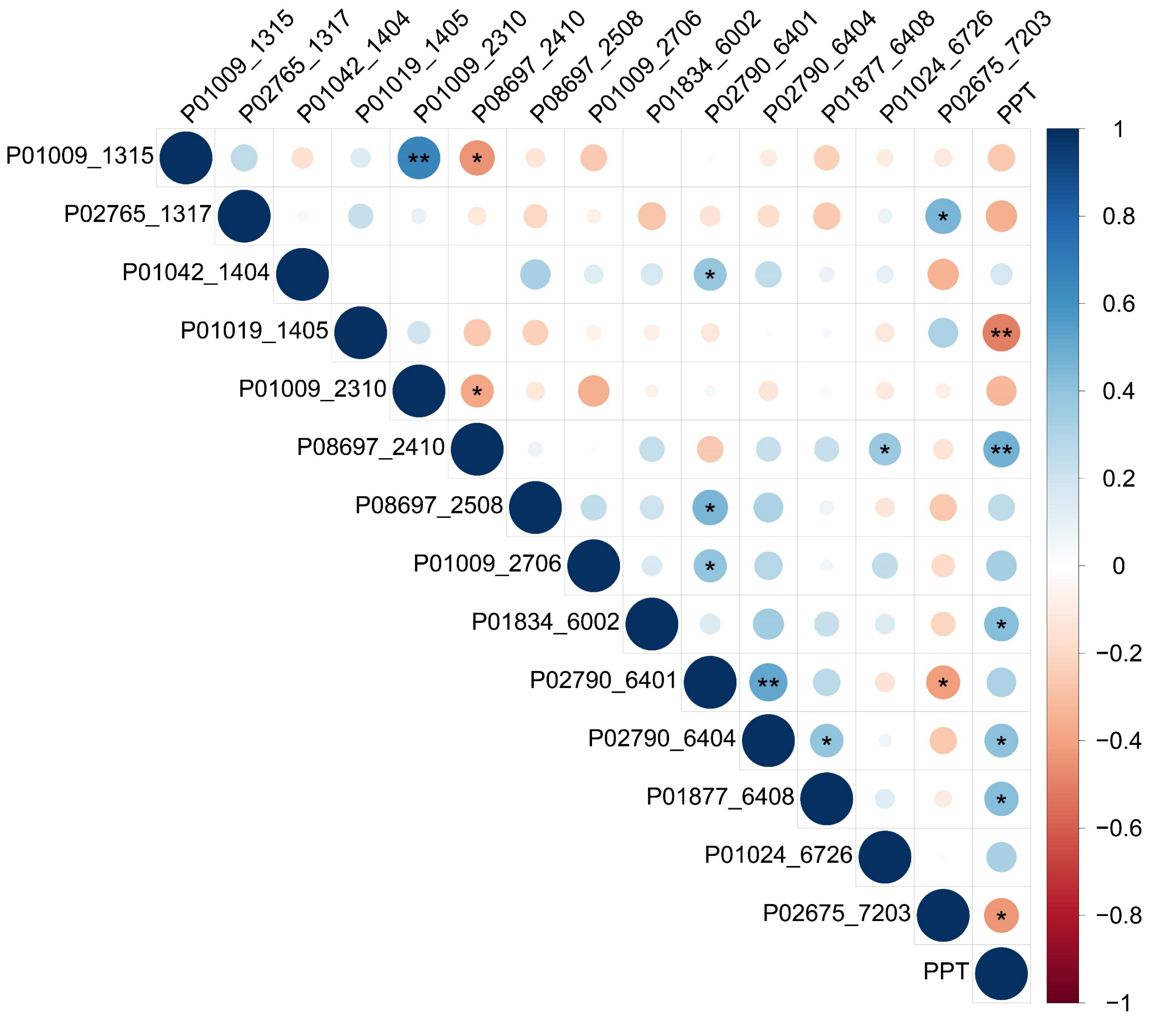

Supplementary Figure S4. Correlation matrix of HADS and significant proteoforms in FM. Spearman correlation matrix of HADS and significant proteoforms from the OPLS model of HADS in FM. Seventeen proteoforms had a significant correlation with HADS. The higher color intensity, the stronger correlation. Red = negative correlation, blue = positive correlation. Variable names are protein accession number and protein spot number, which are equal to presented number in Figure 5 and Table 5, and Supplementary Table S1 and Figure S1. * = p<0.05, ** = p<0.01, HADS = hospital anxiety and depression scale.

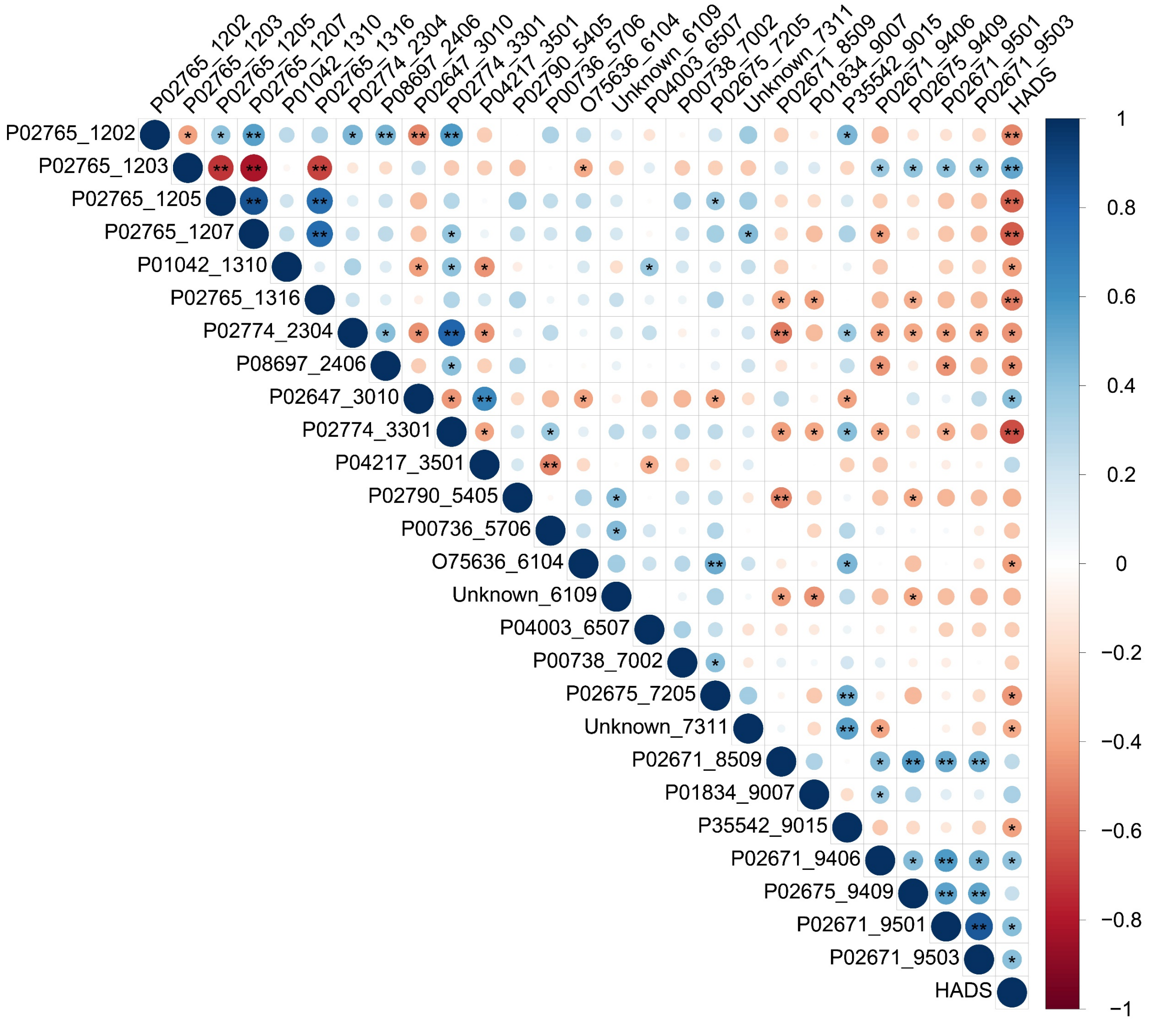

Supplement: Supplementary file 1 — Supplementary information [file 41598_2020_69422_MOESM1_ESM.pdf]
